# Mutant p53-microRNA-200c-ZEB2-Axis-Induced CPT1C Elevation Contributes to Metabolic Reprogramming and Tumor Progression in Basal-Like Breast Cancers

**DOI:** 10.3389/fonc.2022.940402

**Published:** 2022-07-21

**Authors:** Chen-Yun Wang, Cing-Hong Wang, Ru-Tsun Mai, Ting-Wen Chen, Chia-Wei Li, Chi-Hong Chao

**Affiliations:** ^1^ Institute of Molecular Medicine and Bioengineering, National Yang Ming Chiao Tung University, Hsinchu, Taiwan; ^2^ Center For Intelligent Drug Systems and Smart Bio-devices (IDS^2^B), National Yang Ming Chiao Tung University, Hsinchu, Taiwan; ^3^ Department of Biological Science and Technology, National Yang Ming Chiao Tung University, Hsinchu, Taiwan; ^4^ Institute of Bioinformatics and Systems Biology, National Yang Ming Chiao Tung University, Hsinchu, Taiwan; ^5^ Institute of Biomedical Sciences, Academia Sinica, Taipei, Taiwan

**Keywords:** mutant p53, tumor progression, CPT1C, FAO, basal-like breast cancer

## Abstract

*TP53* is mutated in more than 80% of basal-like breast cancers (BLBCs). BLBCs with *TP53* mutation are usually high-grade and have worse responses to chemotherapy, leading to poor clinical outcomes. Wild-type p53 (WTp53) is well-accepted to promote fatty acid oxidation (FAO); however, in this study, we demonstrate that mutant p53 (Mutp53) enhances FAO activity through constitutively upregulating CPT1C *via* dysregulating the miR-200c-ZEB2 axis. Sustained CPT1C expression contributes to the metabolic preference of FAO, epithelial-mesenchymal transition (EMT) phenotypes, migration, invasion, and cancer stemness in BLBC, which is mediated by modulating the redox status. Furthermore, interference of CPT1C expression impairs tumor growth and pulmonary colonization of BLBC cells *in vivo*, and even postpones the occurrence of spontaneous metastasis, resulting in a prolonged disease-specific survival (DSS). Consistently, clinical validation reveals that high CPT1C is observed in breast cancer patients with metastasis and is correlated with poor overall, disease-free, progression-free, and disease-specific survival in BLBC patients. Together, unlike WTp53 which transiently transactivates CPT1C, Mutp53 provides long-term benefits through sustaining CPT1C expression by disturbing the miR-200c-ZEB2 axis, which potentiates FAO and facilitates tumor progression in BLBC, suggesting that targeting Mutp53-CPT1C-driven metabolic reprogramming is promising to serve as novel therapeutic strategies for BLBC in the future.

## Introduction

*TP53* gene, the most frequently mutated tumor suppressor gene (1), is mutated in more than 50% of human cancers (2). Unlike other tumor suppressor genes, over 70% of cancer-associated *TP53* mutations are missense mutations mainly located in the DNA-binding domain (3), causing single amino acid substitutions and consequently DNA-contact or structural mutations (4). *TP53* mutations not only result in loss of function and dominant-negative effect over wild-type p53 (WTp53) but also endow mutant p53 (Mutp53) with gain-of-function properties, which have high relevance to tumorigenesis (5). In breast cancers, the frequency of *TP53* mutation ranges from 12% in luminal A to over 80% in basal-like breast cancers (BLBCs) (2). BLBC comprises 15-20% of breast cancers but harbors the worst clinical outcome as well as high metastasis and tumor recurrence rate (6) due to the lack of targeted therapies. BLBCs with *TP53* mutation are highly invasive, poorly differentiated, usually high-grade, and have a poor response to chemotherapy (7), reflecting the unusually high frequency of *TP53* mutation and the indispensable role of Mutp53 in BLBC.

Beyond the guardian of the genome, p53 has emerged as an important regulator of multiple metabolic pathways in the past decades. p53 is known to suppress the Warburg effect, which is the metabolic alteration favored by a majority of cancer cells, by directly inhibiting the transcription of glucose transporters and several key enzymes involved in glycolysis (8), whereas p53 mutation stimulates the Warburg effect (9). Additionally, p53 regulates glucose metabolism indirectly through microRNAs (miRNAs). For example, p53 downregulates glycolytic enzymes by transactivating miR-34a (10). Recent evidence further indicates that Mutp53 decreases oxidative phosphorylation (OXPHOS) and increases cancer stemness by downregulating the miR-200c-PCK2 axis in BLBCs (11).

In addition to the regulation of glucose metabolism, p53 has been shown to modulate lipid metabolism. Evidence indicates that WTp53 suppresses fatty acid synthesis (FAS) and promotes fatty acid oxidation (FAO) (12). WTp53 could enhance FAO by directly transactivating *carnitine palmitoyltransferase 1C* (*CPT1C*), the brain isoform of the rate-limiting enzyme of FAO, to promote the survival of murine embryonic fibroblasts under metabolic stress (13). However, the positive role of WTp53 in FAO seems contradictory to the extremely high mutation rate of *TP53* and the metabolic preference of FAO in BLBC, as FAO contributes to tumorigenesis (14, 15), metastasis (16–18), cancer stem cell properties (19), chemo-resistance (19) and tumor recurrence (20) in BLBCs. Therefore, the regulatory role of Mutp53 in FAO and the underlying molecular mechanisms require further investigation.

Here, we demonstrate that, instead of suppressing fatty acid degradation, Mutp53 upregulates CPT1C and potentiates FAO by interfering with the miR-200c-ZEB2 axis. Overexpression of CPT1C not only contributes to increased FAO activity but also induces EMT, enhances migration, invasion, and stemness in mammary epithelial cells. On the contrary, knockdown of CPT1C attenuates migration, invasion, and cancer stemness in Mutp53-overexpressing mammary epithelial cells and human BLBC cells harboring endogenous Mutp53. CPT1C mediates Mutp53-exerted oncogenic events by reducing reactive oxygen species (ROS), which might be modulated by the enhanced FAO activity. Furthermore, our *in vivo* study reveals that interference of CPT1C expression leads to impaired tumor growth, attenuated pulmonary colonization, and postponed occurrence of spontaneous metastasis, conferring a better disease-specific survival, which is coincident with clinical validation that high CPT1C expression is associated with metastasis and poor outcomes in BLBC patients. Overall, the robust evidence presented in this study clarifies the controversial role of Mutp53 in lipid metabolism and clearly elucidates the mechanistic relationship between Mutp53 and Mutp53-driven metabolic reprogramming. More importantly, we uncover novel oncogenic roles of CPT1C contributing to the progression of BLBC, which not only is a breakthrough for establishing the p53 regulatory network of cellular metabolism but also accentuates CPT1C as an extraordinarily potential therapeutic target in tumors overexpressing CPT1C, providing crucial implications for developing urgently needed novel treatment strategies for BLBC.

## Materials and Methods

### Cell Lines and Culture

Human normal mammary epithelial cell line, MCF12A (ATCC Cat# CRL-10782, RRID : CVCL_3744), human basal-like breast cancer cell lines, BT549 (ATCC Cat# HTB-122, RRID : CVCL_1092), MDA-MB-231 (ATCC Cat# HTB-26, RRID : CVCL_0062), and murine breast cancer cell line, 4T1(ATCC Cat# CRL-2539, RRID : CVCL_0125), were purchased from American Type Culture Collection (ATCC) during 2016-2017. Mutp53-expressing MCF12A cells (MCF12A-p53R175H, MCF12A-p53R249S, MCF12A-p53R273H and MCF12A-p53R280K), miR-200c-KO MCF12A (MCF12A-miR-200c-Sg1 and MCF12A-miR-200c-Sg2), cells (MCF12A- p53R273H, MDA-MB-231 and BT549) with ZEB-1, ZEB-2, Bmi1 or Slug knockdown were described in our previous study (11). Cells have been cultured for 1-3 months were discarded, and new cells were recovered from cryopreserved stocks to ensure authentication and avoid possible mycoplasma contamination; therefore, cell authentication and contamination of mycoplasma were not re-examined in our laboratory.

### Plasmid Construction

To construct pCDH/CPT1C, CPT1C cDNA fragments amplified from reverse transcription-PCR were cloned into the pCDH-CMV-MCS-EF1-Puro lentiviral vector. Lentiviral shRNA constructs for knockdown CPT1C were provided by the National RNAi Core Facility services at Academia Sinica in Taiwan. Mutp53-expressing constructs (pLenti6/V5-p53_R175H, RRID : Addgene_22936; pLenti6/V5-p53_R249S, RRID : Addgene_22935; pLenti6/V5-p53_R273H, RRID : Addgene_22934; and pLenti6/V5-p53_R280K, RRID : Addgene_22933) and their control vectors were purchased from Addgene (21). To obtain pLenti6/V5-Luc2, the p53 cDNA fragment in pLenti6/V5-p53_R273H was replaced with a PCR-amplified *Luc2* cDNA fragment. Cloning strategies, sequences of cloning primer sets, and plasmid maps will be provided upon request.

### Generation of Stable Expressed, Knocked-Down, or Knocked-Out Cell Lines

MCF12A, BT549, MDA-MB-231, and 4T1 cells were infected with lentiviral constructs for gene overexpression or knockdown. Infected cells were selected with Puromycin (2 μg/ml) or Blasticidin (10 μg/ml) for two weeks to establish stable clones.

### Total RNA Extraction and Real Time-PCR

The RNA extraction and RT-PCR were performed as described before (11). The expression of CPT1C mRNA was examined by using the primer set: CPT1C-F, TGCCATGTCGTTCCATTCTCCC; CPT1C-R, GCCGACTCATAAGTCAGGCAGA. Actin gene served as an internal control for quantitation using the primer set: ACTB-F, CACCATTGGCAATGAGCGGTTC; ACTB-R, AGGTCTTTGCGGATGTCCACGT.

### Antibodies

The commercial available antibodies and dilutions used in the immunoblotting analysis were listed below: anti-ZEB1 (1:1000, Cell Signaling, Danvers, MA, USA, #3369), anti-ZEB2 (1:1000, Cell Signaling, #97885), anti-p53 (1:2000, Santa Cruz, Dallas, Texas, USA, sc-126), anti-E-Cadherin (1:500, Santa Cruz, sc-8426), anti-N-Cadherin (1:250, Santa Cruz, Sc-59987), anti-CPT1C (1:500, Santa Cruz, sc-514555), and anti-Actin (1:1000, Santa Cruz, sc-47778).

### Mammosphere Formation Assay, Transwell Migration/Invasion Assay, and Soft Agar Foci Formation Assay

Detailed experimental procedures were described in our previous study (11). Briefly, for mammosphere formation assay, 4-5x10^4^ of MCF12A, BT549, or MDA-MB-231 cells were used, and mammosphere numbers were determined after growing in suspension culture for 6-7 days. For migration assay, cells (1x10^5^ for MCF12A, 5x10^4^ for MDA-MB-231, and 5x10^4^ for BT549 cells/well) were seeded into the upper chamber of a transwell. For invasion Assay, cells (2x10^5^ for MCF12A, 5x10^4^ for MDA-MB-231, and 1x10^5^ for BT549 cells/well) were seeded into the upper chamber precoated with 100 μL of 2% Matrigel (Corning, NY, USA). The migrated/invaded cells were then visualized by crystal violet staining after 16-24 hours. To perform soft-agar foci formation assay, cells (2.5x10^4^ for MCF12A and 5x10^4^ for MDA-MB-231 cells/well) were grown in the soft agarose layer (0.4%) on top of the hard agarose (0.8%) in six-well plates (Falcon, Chicago, Illinois, USA). The foci numbers were then determined after 3-4 weeks of incubation. All experiments were performed in triplicate.

### Extracellular Metabolic Flux Analysis

FAO assay was performed by combining XF palmitate-BSA FAO substrate with XF Mito Stress kit (Agilent, Santa Clara, CA, USA). Cells (3.5x10^4^ for MCF12A, and 4x10^4^ for BT549 cells/well) were resuspended in 80 μL of culture medium containing palmitate-BSA conjugate (100 μmol/L) and carnitine (1 mmol/L) and seeded into Seahorse XF Cell Culture Microplates. The next day, cells were washed and the medium was replaced by XF Assay Medium Modified DMEM containing GlutaMAX™ (Billings, Montana, USA), palmitate-BSA conjugate (100 μmol/L), carnitine (1 mmol/L), and distilled water (for vehicle group) or etomoxir (2-4 μmol/L, for ETO group; Cayman Chemical, Ann Arbor, Michigan, USA). After cells were incubated for 1-2 hours at 37°C in a non-CO_2_ incubator, the oxygen consumption rate was measured by Seahorse XF Extracellular Flux Analyzer. All experiments were performed in triplicate. The results were analyzed using the software XFp Wave. The basal ratio was calculated as [(vehicle basal–ETO basal respiration)/vehicle basal respiration] x 100%.

### Determination of ATP Level

To determine the FAO-associated ATP production, cells were cultured in a glucose-free medium (containing carnitine and GlutaMAX™) supplemented with/without palmitate-BSA conjugate (100 μmol/L) in the absence/presence of etomoxir (2 μmol/L) for 2 hours then subjected to the measurement of ATP level. ATP level was measured by using the ATP Determination kit (Invitrogen, Waltham, MA, USA) following the manufacturer’s instructions. Briefly, cell pellets were lysed by Bioruptor plus sonication device (Diagenode, Denville, NJ, USA), and sample assays were prepared (10% of sample solution mixed with 90% of reaction solution) followed by incubation in the dark for 10 minutes. The luminescence was measured by the CLARIOstar microplate reader (BMG LABTECH, Ortenberg, Germany). All experiments were performed in triplicate and normalized to respective protein concentrations.

### Determination of Total Cellular ROS Level

To determine the cellular ROS level, cells grown to confluence were stained with CellROX™ Green Reagent (Invitrogen) at a final concentration of 2.5 μmol/L and incubated for 30 minutes at 37°C for MCF12A, and 10 μmol/L with 1-hour incubation at 37°C for MDA-MB-231, which were then washed with PBS, trypsinized, and subjected to FACS analysis by NovoCyte Flow Cytometer (ACEA Biosciences, San Diego, CA, USA). On the other hand, cells in suspension were stained with DCFDA/H2DCFDA (Abcam, Cambridge, UK) at a final concentration of 1 μmol/L and incubated for 15 minutes at 37°C for MCF12A, and 4 μmol/L with 15-minute incubation at 37°C for MDA-MB-231, which were then washed and subjected to FACS analysis.

### Determination of NADPH/NADP^+^ Ratio by Metabolome Analysis

Detailed information on metabolome analysis was described in our previous study (11). Briefly, the quantification of 116 metabolites including NADPH and NADP^+^ was conducted by the C-SCOPE service of [Human Metabolome Technologies, Inc., (HMT)] using capillary electrophoresis time-of-flight mass spectrometry (CE-TOFMS, Agilent CE-TOFMS system Machine No. 3, Agilent Technologies) for cation analysis and CE- tandem mass spectrometry (CE-QqQMS; CE system with Agilent 6460 TripleQuad LC/MS Machine No, QqQ1, Agilent Technologies) for anion analysis. To prepare the sample for metabolome analysis, cells were washed with 5% mannitol solution, and cellular metabolites were extracted by methanol. Extracts were added with internal standards and then filtered to remove macromolecules. Filtrates were then subjected to metabolomic analysis at HMT. Detailed methods will be provided upon request. NADPH/NADP^+^ ratio was calculated as following: [NADPH]/[NADP^+^].

### *In Vivo* Studies

For orthotopic inoculation, female SCID mice (NOD.CB17-*Prkdc^scid^*/JNarl; purchased from National Laboratory Animal Center, Taipei, Taiwan; 8-10 weeks old) were inoculated with 3x10^6^ of MDA-MB-231-Control and siCPT1C cells mixed with Matrigel (Matrigel: PBS=1:1) into the second and the fourth mammary fat pads. Tumor size was measured every three days with a caliper, and tumor volume was determined with the formula: (d1x d2^2^)/2 (d1: larger diameter; d2: smaller diameter). For tail vein injection, female SCID mice (8-10 weeks old) were randomly divided into two groups. 1x10^6^ of MDA-MB-231-Control or siCPT1C cells expressing firefly luciferase were suspended in 100 μL of PBS and injected into tail veins. Metastasis was monitored by the *in vivo* imaging system (IVIS) followed by intraperitoneal injection (IP) of D-luciferin (150 mg/kg body weight). Briefly, the images were captured by using Auto exposure, then the raw signal was calibrated, and the measurement region of interests (ROIs) were determined. Animal experiments and animal care were handled according to the protocol approved by the Institutional Animal Care and Use Committee at National Yang Ming Chiao Tung University, Taiwan. (IACUC number: NCTU-IACUC-106030).

### TCGA Data Processing and Survival Analysis

The expression levels of miRNAs/mRNA, mutation profiles, and clinical information for the TCGA Breast invasive carcinoma cohort (BRCA) were downloaded from Broad GDAC Firehose (https://gdac.broadinstitute.org). The mRNA expression values calculated with RSEM (22) were used for the PAM50 model analysis. All the breast cancer samples were classified into four breast cancer subtypes, i.e., basal-like breast tumor, HER2-enriched, Luminal A, and Luminal B (23, 24). *TP53* mutated patients are defined as those carrying non-silent mutations on *TP53*. All four synonymous (silent) mutations found on *TP53* were predicted to be likely benign by InterVar (25), hence treated as non-mutated in our analysis.

To perform survival analysis according to CPT1C expression level, all the breast cancer patients were divided into two equal-sized groups based on the expression level of CPT1C. The survival information for OS (overall survival), DFS (disease-free survival), PFS (progression-free survival), and DSS (disease-specific survival) were all downloaded from cBioPortal (26). Kaplan-Meier survival plot (KM plot) and log-rank test were used for the comparison of survival data between the high expression and low expression groups.

### Data and Materials Availability

All data files supporting the findings of this study are available upon reasonable request.

## Results

### Mutant p53 Enhances Fatty Acid β-Oxidation Activity in Immortal Mammary Epithelial Cells

**Figure 1:**
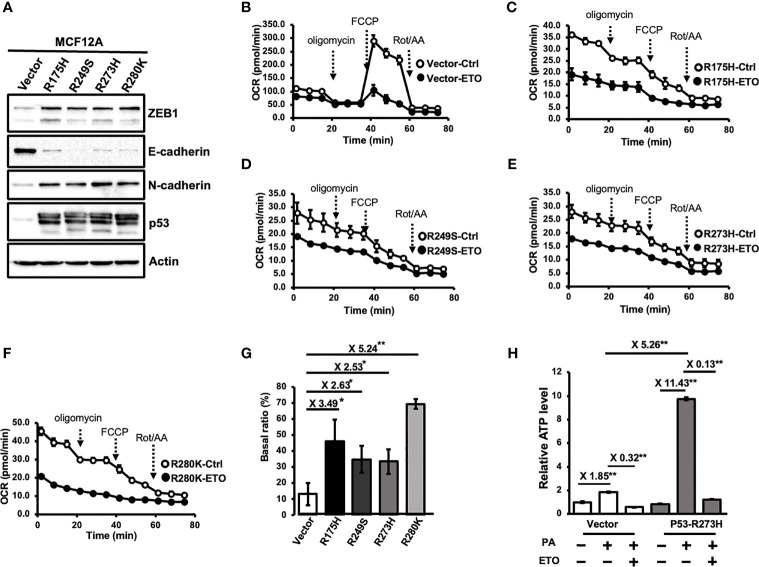
Mutant p53 enhances fatty acid beta-oxidation (FAO) activity in immortal mammary epithelial cells. **(A)** Stable expression of p53 hot-spot mutations in MCF12A cells. The expressional levels of ZEB1, E-Cadherin, N-Cadherin, p53, and Actin were examined by Western Blotting. All of the immunoblotting analysis was performed under the same experimental conditions. Results were obtained from the same or different PAGEs using the same samples and presented as cropped images. **(B–G)** Mutant p53 enhances FAO activity. FAO activity of MCF12A-Ctrl **(B)**. MCF12A-p53^R175H^ **(C)**. MCF12A-p53^R249S^ **(D)**. MCF12A-p53^R273H^ **(E)** and MCF12A-p53^R280K^ **(F)** were measured by seahorse metabolic flux analyzer with FAO assay kit. **(G)** the FAO activity of individual cell lines was determined by averaging Basal ratios from three independent experiments (mean ± SD, n = 3). Basal ratio = (basal OCR^Ctrl^- basal OCR^ETO^)/basal OCR^Ctrl^. The two sides of the lines above the bars indicated the comparison of each Mutp53 to the control, and the value of fold changes with the asterisks representing significance was marked above the line. Data were analyzed by unpaired t-test. *p < 0.05; **p < 0.01. **(H)** FAO-produced ATP is increased by the expression of p53 mutant. MCF-12A-Ctrl (Vector) and MCF12A-P53-R273H cells (P53-R273H) were treated with palmitate and/or ETO (2 μM) and then subjected to the ATP assay to measure the steady-state ATP level (mean ± SD, n = 3). The relative ATP level was plotted by comparing to the ATP level of the Vector group without the treatment of PA. The two sides of the lines above the bars indicated the comparison of the two groups, and the value of fold changes with the asterisks representing significance was marked above the line. Data were analyzed by unpaired t-test. **p < 0.01.

To investigate whether Mutp53 involves in the regulation of FAO, a set of p53 hot-spot mutations-bearing MCF12A cell lines was used (11, 21). Consistent with previous findings, MCF-12A cells bearing p53 mutations (R175H, R249S, R273H, and R280K) showed an EMT phenotype (**Figure 1A**). Overexpression of p53 mutations also significantly enhanced stemness (**Supplementary Figures S1A, B**). and led to the transformation of mammary epithelial cells (**Supplementary Figure S1C**). To examine whether these p53 hot-spot mutant cells exhibit elevated FAO activity, we performed a non-isotopic FAO assay by measuring the FAO-associated oxygen consumption rate (OCR) with Seahorse XF Extracellular Flux Analyzer (**Figures 1B–F**) to evaluate the ability of oxidizing fatty acids (27). The basal OCR between the vehicle control and the etomoxir (ETO)-treated group in the presence of palmitate was compared to determine the FAO-mediated oxygen consumption. To quantify the FAO activity which contributes to total cellular oxygen consumption, a “basal ratio” (the ratio between FAO-mediated OCR and total OCR) was assigned by the following equation: [(basal respiration of the vehicle group–basal respiration of the ETO group)/basal respiration of the vehicle group] x 100%. As shown in **Figure 1G**, the basal ratio of control cells is around 13%, which means FAO contributes to 13% of cellular oxygen consumption, whereas it was at least doubled in Mutp53-bearing MCF12A cells. Additionally, palmitate feeding exerted a more profound effect in fueling ATP production in p53 mutant-expressing cells, as an 11.43- and 1.85-fold increase of steady-state ATP level was observed in MCF12A-p53^R273H^ and control cells, respectively, and this increase was abolished by ETO-treatment (**Figure 1H**), indicating an increased FAO activity in p53 mutant-expressing cells. Together, all these results suggested that p53 mutations could upregulate cellular FAO activity in mammary epithelial cells.

### Etomoxir Treatment Interferes With Mutp53-Induced Biological Effects

Since Mutp53 has been shown to affect cancer migration, invasion, and cancer stemness in addition to cell proliferation (28), to understand whether FAO is involved in Mutp53-promoted tumor properties, we suppressed cellular FAO activity with etomoxir (ETO), the pan-CPT1 inhibitor. The p53-R273H and p53-R280K MCF-12A cells were pretreated with ETO (50 and 100 μmol/L) for seven days, then subjected to migration, invasion, and mammosphere forming assays in the absence of ETO to examine whether interfering with Mutp53-induced FAO could compromise Mutp53-enhanced cellular motility and stemness. Pretreatment of ETO significantly decreased both migration (**Supplementary Figure S2A**) and invasion (**Supplementary Figure S2B**) ability, as well as sphere formation (**Supplementary Figure S2C**) without continuous treatment of ETO. Similar effects were also observed when ETO (50 and 200 μmol/L) was administrated during assays in p53-R273H MCF-12A and BT549 cells (**Supplementary Figures S2A–C**). Moreover, ETO treatment resulted in a dose-dependent inhibition on the foci formation of p53-R273H and p53-R280K MCF-12A cells in soft agar (**Supplementary Figure S2D**), suggesting inhibition of FAO disturbs the Mutp53-mediated anchorage-independent growth.

Taken together, these results indicated that ETO treatment interferes with Mutp53-induced migration, invasion, stemness, and cell transformation, implying a crucial role of FAO in Mutp53-mediated biological effects in BLBCs.

### Mutant p53 Activates FAO Activity Through Upregulating CPT1C

**Figure 2:**
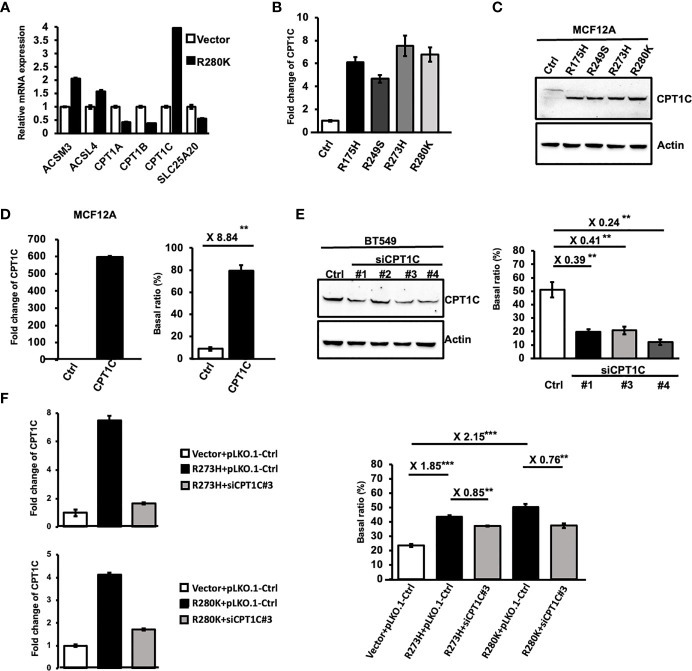
Mutant p53 activates FAO activity through upregulating CPT1C. **(A)** The differential expression of lipid metabolism-related genes induced by mutant p53. The relative mRNA expression level of *ACSM3*, *ACSL4*, *CPT1A*, *CPT1B*, *CPT1C*, and *SLC25A20* between control and MCF12A-p53^R280K^ cells were determined by Q-PCR (mean ± SD, n = 3). **(B)** The mRNA expression of *CPT1C* is upregulated by p53 hot-spot mutants. Relative mRNA expression of *CPT1C* between control cells and p53 mutant-MCF12A cells was determined by Q-PCR (mean ± SD, n = 3). **(C)** Protein expression of CPT1C is upregulated by p53 hot-spot mutants. The expressional levels of CPT1C and Actin in control (Ctrl) and mutant p53-bearing MCF12A cells were examined by Western Blotting. **(D)** The FAO activity is upregulated by CPT1C overexpression in mammary epithelial cells. Left: The expressional levels of CPT1C and Actin in control (Ctrl) and CPT1C-overexpressing MCF12A (CPT1C) cells were examined by Western Blotting. Right: The FAO activity of Ctrl and CPT1C cells was presented as a Basal ratio and shown as a bar graph (mean ± SD, n = 3). **(E)** The FAO activity is decreased by the knockdown of CPT1C in BLBC cells. Left: The expressional levels of CPT1C and Actin in control (Ctrl) and CPT1C-KD BT549 cells (siCPT1C#1, #3, and #4) were examined by Western Blotting. Right: The FAO activity of Ctrl and CPT1C cells was presented as a Basal ratio and shown as a bar graph (mean ± SD, n = 3). The two sides of the lines above the bars indicated the comparison of each KD clone to the control, and the value of fold changes with the asterisks representing significance was marked above the line. Data were analyzed by unpaired t-test. **p < 0.01. **(F)** Downregulating CPT1C expression interferes with p53 mutant-enhanced FAO activity. Left: The mRNA expressional level of *CPT1C* in control and CPT1C-KD MCF12A-p53^R273H^ and MCF12A-p53^R280K^ cells (siCPT1C#3) were examined by Q-PCR. Right: The FAO activity of Ctrl (Vector+pLKO.1), MCF12A-p53^R273H^ (p53^R273H^+pLKO.1), CPT1C-KD MCF12A-p53^R273H^ (p53^R273H^+siCPT1C#3), MCF12A-p53^R280K^ (p53^R280K^+pLKO.1), CPT1C-KD MCF12A-p53^R280K^ (p53^R280K^+siCPT1C#3) cells were presented as Basal ratio and shown as a bar graph (mean ± SD, n = 3). The two sides of the lines above the bars indicated the comparison of the two groups, and the value of fold changes with the asterisks representing significance was marked above the line. Data were analyzed by unpaired t-test. **p < 0.01; ***p<0.001.

To further elucidate the molecular mechanisms accounting for Mutp53-induced FAO, the mRNA expression of several FAO-associated genes was examined by Q-PCR in MCF12A-p53-R280K cells (**Figure 2A**). Among these enzymes, carnitine palmitoyltransferase 1C (CPT1C), the brain-specific isoform of the rate-limiting enzyme of FAO, was identified as one of the targets significantly upregulated by Mutp53^R280K^. Moreover, this enhanced CPT1C expression was also found in MCF12A cells expressing other p53 hot-spot mutations in both mRNA (**Figure 2B**) and protein level (**Figure 2C**), implying a putative role of CPT1C in Mutp53-enhanced FAO.

Although the acyl-carnitine activity of CPT1C has been confirmed by *in vitro* biochemical analyses (29), whether CPT1C contributes to cellular FAO activity is still under debate (30, 31). Hence, we first overexpressed CPT1C in MCF-12A cells (**Figure 2D** left) and performed an FAO assay to address this issue. As shown in (**Figure 2D** right), overexpression of CPT1C led to an 8.84-fold increase in the basal ratio, indicating cellular FAO activity was enhanced by CPT1C in mammary epithelial cells. Moreover, the observation that interference of CPT1C expression attenuated FAO activity in BLBC cells (from 51.05% to 12.04%; **Figure 2E**; **Supplementary Figures S3A, B**) further supported this notion. Since our results clearly indicated that CPT1C expression contributes to the FAO activity in mammary epithelial and BLBC cells, we compromised the enhanced CPT1C expression by RNA interference in p53-R273H and p53-R280K expressing MCF-12A cells (**Figure 2F** left, **Supplementary Figures S3C, D**) to address whether CPT1C is responsible for Mutp53-enhanced FAO activity. As shown in **Figure 2F** and **Supplementary Figure S3E**, similar trends were observed in CPT1C-KD p53-R273H- and p53-R280K-MCF-12A cells. The basal ratio decreased from 43.59% to 37.2% in p53-R273H and from 50.61% to 38.35% in p53-R280K MCF-12A cells (**Figure 2F** right). All these results indicated that Mutp53 enhances FAO by upregulating the expression of CPT1C.

### CPT1C Induces EMT, Enhances Migration, Invasion, and Stemness in Mammary Epithelial Cells and Contributes to Mutp53-Mediated Biological Effects in Cancer Progression

Accumulated evidence indicates that enhanced FAO not only promotes cancer cell survival under metabolic stress but also enhances tumor progression by inducing EMT and cancer stemness (32). In viewing that CPT1C plays a causal role in cell proliferation and survival (30), we further examined whether CPT1C expression also facilitates cancer progression by inducing EMT-associated phenotypes and therefore contributes to Mutp53-mediated biological effects.

**Figure 3:**
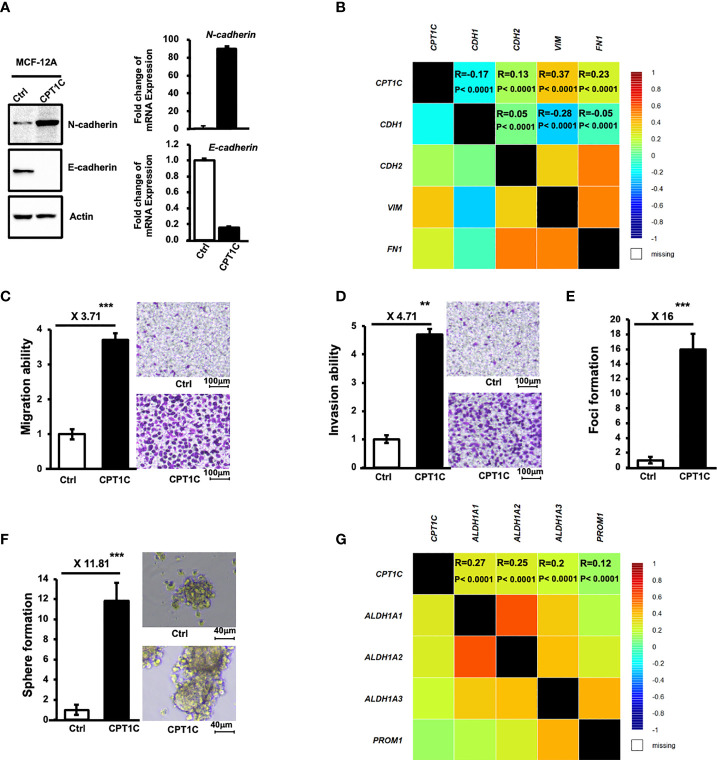
CPT1C induces EMT and enhances migration, invasion, and stemness in mammary epithelial cells. **(A)** Ectopic expression of CPT1C induces EMT in mammary epithelial cells. The expressional levels of N-Cadherin, E-Cadherin, and Actin in control (Ctrl) and CPT1C-overexpressing (CPT1C) MCF12A cells were examined by Western Blotting. Relative mRNA expression of *N-cadherin* and *E-cadherin* between control cells and CPT1C-overexpressing (CPT1C) MCF12A cells were determined by Q-PCR (mean ± SD, n = 3). **(B)** CPT1C shows a slight to moderate positive association with EMT in breast cancer patients. Correlation map showing the Pearson’s pairwise correlations among CPT1C, CDH1 (E-cadherin), CDH2 (N-cadherin), VIN (vimentin), and FN1 (fibronectin) in breast cancer patients (N = 4712). Each cell represents a statistical relation between two genes and is colored according to the value of the Pearson correlation coefficient ranging from dark blue (coefficient = -1) to dark red (coefficient = 1). R: correlation coefficient value; P: corresponding p-value. Results are derived from bc-GeneExMiner v4.5 (http://bcgenex.ico.unicancer.fr). **(C, D)** Ectopic expression of CPT1C enhances cell migration **(C)** and invasion **(D)** Migration and invasion ability of CPT1C-overexpressing MCF12A cells were analyzed by migration and invasion assay. The relative migration/invasion ability is presented as a fold change of the numbers of cells passed through trans-wells between control and CPT1C groups (mean ± SD; n=3). The two sides of the lines above the bars indicated the comparison of CPT1C-overexpressing cells to the control cells, and the value of fold changes with the asterisks representing significance was marked above the line. Data were analyzed by unpaired t-test. **p <0.01; ***p < 0.001. Representative micrographs of migrated **(C)** or invaded **(D)** cells were shown on the right. **(E)** CPT1C overexpression enhances anchorage-independent growth. A soft-agar foci formation assay was performed to determine the anchorage-independent growth ability of CPT1C-overexpressing MCF12A cells. Foci number in triplicate dishes was counted and presented as fold change between control and CPT1C groups (mean ± SD; n = 3). The two sides of the line above the bars indicated the comparison of CPT1C-overexpressing cells to the control cells, and the value of fold changes with the asterisks representing significance was marked above the line. Data were analyzed by unpaired t-test. ***: p < 0.001. **(F)** Overexpression of CPT1C enhances stemness. CPT1C-overexpressing MCF12A cells exhibit higher mammosphere forming ability. Control or CPT1C-overexpressing MCF12A cells were grown in suspension culture for 7-8 days to form mammospheres. Mammospheres with a diameter larger than 40 μm were counted and presented as fold change. Results were derived from experiments done in triplicate (mean ± SD; n = 3). The two sides of the line above the bars indicated the comparison of CPT1C-overexpressing cells to the control cells, and the value of fold changes with the asterisks representing significance was marked above the line. Data were analyzed by unpaired t-test. ***p < 0.001. Representative micrographs of mammospheres under a microscope are shown on the right. **(G)** CPT1C shows a slight to moderate positive association with stemness markers in breast cancer patients. Correlation map showing the Pearson’s pairwise correlations among CPT1C and stemness markers, ALDH1A1, ALDH1A2, ALDH1A3, and CD133 (PROM1) in breast cancer patients. R: correlation coefficient value; P: corresponding p-value. Results are derived from bc-GeneExMiner v4.5 (http://bcgenex.ico.unicancer.fr).

To investigate the role of CPT1C in the progression of BLBC, MCF12A cells overexpressing CPT1C were subjected to a series of analyses to examine whether CPT1C contributes to EMT, migration, invasion, and stemness. As shown in **Figure 3A**, overexpression of CPT1C resulted in an increased expression of N-Cadherin and a decreased expression of E-cadherin in mammary epithelial cells. This EMT phenotype induced by CPT1C *in vitro* was further supported by an *in vivo* observation as a slight to moderate positive correlation between CPT1C and the EMT markers (CPT1C vs. E-Cadherin, R=-0.17, P<0.0001; CPT1C vs. N-Cadherin, R=0.13, P<0.0001; CPT1C vs. Vimentin, R=0.37, P<0.0001; CPT1C vs. Fibronectin, R=0.23, P<0.0001; **Figure 3B**) was found in breast cancer patients. High expression of CPT1C also led to a significant enhancement in migration (**Figure 3C**) and invasion (**Figure 3D**) potential, as well as anchorage-independent growth (**Figure 3E**). Additionally, CPT1C greatly increased mammosphere forming ability (**Figure 3F**). The mRNA level of *CPT1C* in mammospheres was highly elevated when compared with 2D culture in MCF-12A-p53^R273H^, BT549, and MDA-MB-231 cells (**Supplementary Figure S4**). Moreover, the expression of CPT1C was positively correlated with several CSC markers (CPT1C vs. ALDH1A1, R=0.27, P<0.0001; CPT1C vs. ALDH1A2, R=0.25, P<0.0001; CPT1C vs. ALDH1A3, R=0.2, P<0.0001; CPT1C vs. PROM1, R=0.12, P<0.0001; **Figure 3G**) in breast cancer patients, further suggesting an important role of CPT1C in maintaining cancer stemness. Together, all these results indicated that CPT1C might promote BLBC progression by inducing cell transformation, strengthening stem cell properties, and enhancing cellular motility and invasiveness through the induction of EMT.

**Figure 4:**
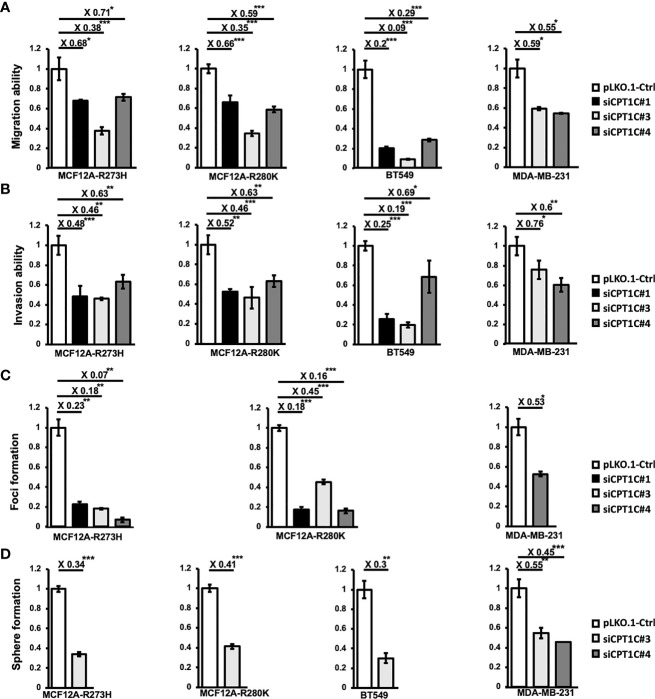
Interference of CPT1C expression attenuates mutant p53-mediated biological effects. CPT1C expression was downregulated by RNA interference in mutant p53-overexpressing cells (MCF12A-p53^R273H^ and MCF12A-p53^R280K^) and BLBC cell lines (BT549 and MDA-MB-231), and these cell lines were then subjected to the following analysis. **(A, B)** Downregulating CPT1C expression attenuates migration and invasion ability in mutant p53-overexpressing mammary epithelial cells and BLBCs. The migration **(A)** and invasion **(B)** ability of CPT1C-KD cells (siPCK2 #1, #3, or #4) were analyzed by migration/invasion assay done in triplicate. The relative migration/invasion ability is presented as a fold change of the numbers of cells passed through trans-wells between control and CPT1C-KD cells (mean ± SD; n = 3). The two sides of the lines above the bars indicated the comparison of each KD clone to the control, and the value of fold changes with the asterisks representing significance was marked above the line. Data were analyzed by unpaired t-test. *p <0.05; ***p < 0.001. **(C)** Downregulating CPT1C expression diminishes the anchorage-independent growth in MDA-MB-231 and mutant p53-overexpressing mammary epithelial cells. The anchorage-independent growth ability of CPT1C-KD cells was examined by soft-agar foci formation assay (mean ± SD; n = 3). The two sides of the lines above the bars indicated the comparison of each KD clone to the control, and the value of fold changes with the asterisks representing significance was marked above the line. Data were analyzed by unpaired t-test. *p <0.05; **p <0.01; ***p < 0.001. **(D)** Interference of CPT1C expression suppresses stemness/cancer stemness in mutant p53-overexpressing mammary epithelial cells and BLBCs. The stem cell/cancer stem cell properties of CPT1C KD cells were examined by mammosphere forming assay (mean ± SD; n = 3). The two sides of the lines above the bars indicated the comparison of each KD clone to the control, and the value of fold changes with the asterisks representing significance was marked above the line. Data were analyzed by unpaired t-test. **p < 0.01; ***p < 0.001.

To elucidate whether CPT1C contributes to Mutp53-mediated oncogenic events, CPT1C-KD Mutp53-overexpressing MCF12A (**Supplementary Figures S3C, D**) and two human BLBC cell lines-BT549 (**Figure 2E**) and MDA-MB-231 (**Supplementary Figure S5**) with intrinsic p53 mutations were established and subjected to migration, invasion, soft-agar foci formation, and mammosphere forming assays. Downregulating CPT1C expression not only attenuated Mutp53-enhanced migration (**Figure 4A**, left) and invasion (**Figure 4B**, left) abilities but also dramatically suppressed Mutp53-induced foci formation (**Figure 4C**, left) in the Mutp53-overexpressing MCF-12A cells. Moreover, knockdown of CPT1C compromised the Mutp53-enhanced sphere formation ability (**Figure 4D**, left). Since similar effects were seen in BLBC cell lines, BT549 and MDA-MB-231 (**Figures 4A–D** right), all of our results indicated that CPT1C activated by Mutp53 not only involves in p53 mutation-enhanced FAO activity but also contributes to Mutp53-induced malignant properties. We suggested that CPT1C might play a critical role in regulating tumor cell motility, invasiveness, transformation, and maintenance of cancer stem cell properties in BLBC.

**Figure 5:**
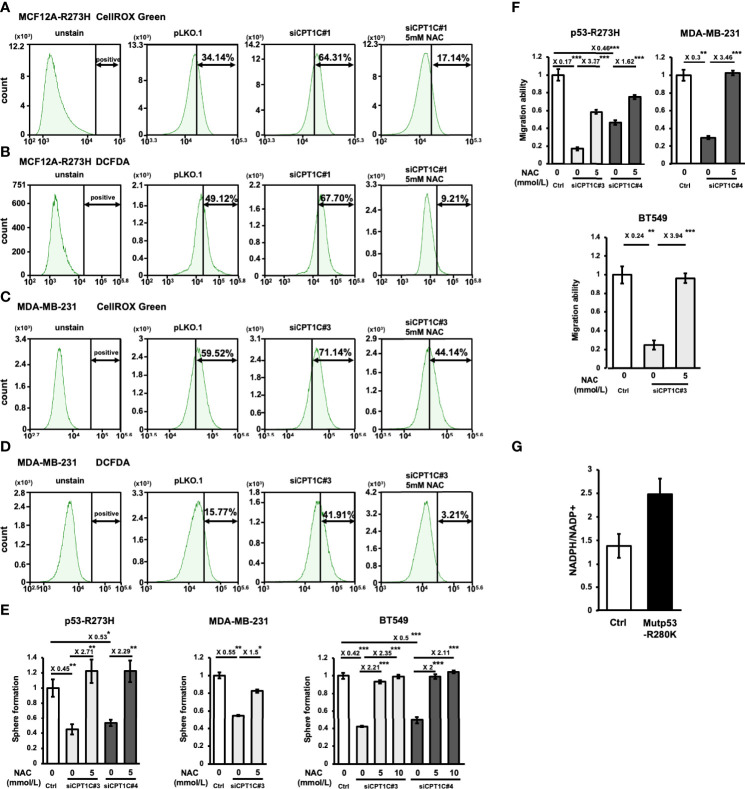
CPT1C regulates mutant p53-mediated biological effects through modulating redox status. **(A–D)** Total cellular ROS level is elevated by knockdown of CPT1C in MCF12A^R273H^ **(A, B)** and MDA-MB-231 **(C, D)** cells and is compromised by N-acetyl cysteine (NAC) treatment. Cells were stained with CellROX™ Green Reagent **(A, C)** or DCFDA **(B, D)**, then the fluorescence was measured by FACS to determine the total cellular ROS. Similar trends in the representative images were observed in three independent experiments. **(E, F)** CPT1C deficiency-mediated suppression of cancer stemness and migration ability are rescued by NAC treatment. MCF12A^R273H^ [**(E**, **F)**, left], MDA-MB-231 [**(E**, **F)** middle], and BT549 [**(E, F)** right] cells treated with NAC (0, 5, or 10 mmol/L) for 1 day and its Ctrl cells were subjected to mammosphere formation assay and transwell migration assay (mean ± SD; n = 3). The two sides of the lines above the bars indicated the comparison of the two groups, and the value of fold changes with the asterisks representing significance was marked above the line. Data were analyzed by unpaired t-test, *p < 0.05, **p < 0.01, ***p < 0.001. **(G)** The ratio of NADPH/NADP^+^ is upregulated by mutant p53. The absolute amount of NADPH and NADP^+^ were measured by CE-TOFMS and CE-QqQMS analysis, and the ratio of NADPH/NADP^+^ in MCF12A-ctrl and MCF12A-p53^R280K^ were compared and shown in bar graphs (mean ± SD, n = 2).

Since FAO contributes to the generation of cytosolic NADPH, which is important for cancer cells to counteract oxidative stress (33), and a previous study revealed that CPT1A-driven FAO decreases ROS levels to avoid anoikis in colorectal cancer (CRC) cells (34), we hypothesized that Mutp53-CPT1C-enhanced FAO activity is critical for maintaining redox homeostasis in BLBC. Examination of the cellular ROS levels in CPT1C-KD p53-R273H-MCF12A (**Figures 5A, B**) and MDA-MB-231 (**Figures 5C, D**) cells, respectively, by using two independent reagents, CellROX™ Green Reagent (**Figures 5A, C**) and DCFDA (**Figures 5B, D**), revealed that knockdown of CPT1C led to increased ROS levels in both cell lines, whereas treatment of the antioxidant, N-acetyl cysteine (NAC), compromised the elevation of cellular ROS, which supported our previous hypothesis. To further examine whether CPT1C promotes BLBC progression by reducing ROS production, CPT1C-KD p53-R273H MCF12A, MDA-MB-231, and BT549 cells were pretreated with 5 or 10 mmol/L of NAC for 1 day and subjected to mammosphere forming assay and transwell migration assay. As shown in **Figure 5E**, treatment of NAC rescued the suppression of sphere-forming ability caused by the knockdown of CPT1C. Moreover, NAC treatment counteracted CPT1C knockdown-reduced migration ability (**Figure 5F**). Furthermore, higher NADPH/NADP^+^ was observed in Mutp53-bearing MCF12A (MCF12A^R280K^) cells (**Figure 5G**), suggesting CPT1C-mediated FAO might provide a NADPH pool for tumor cells, which is used to counteract oxidative stress. These results suggested that CPT1C exerts Mutp53-mediated biological effects through modulating cellular redox status.

### Mutant p53 Activates CPT1C Expression Through the miR-200c-ZEB2 Axis

**Figure 6:**
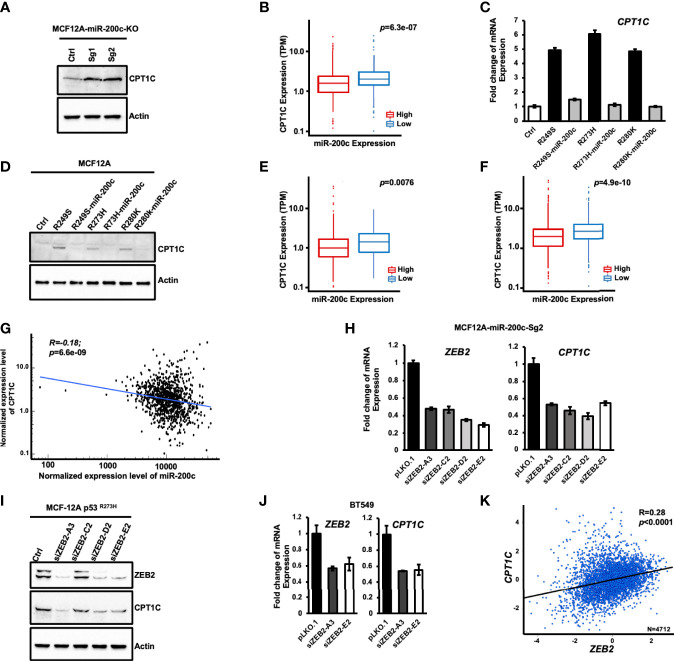
Mutant p53 activates CPT1C expression through the miR-200c-ZEB2 axis. **(A)** miR-200c-deficiency leads to enhanced expression of CPT1C in mammary epithelial cells. The expression of CPT1C and Actin in control (Ctrl) and miR-200c-KO MCF12A cells (MCF12A-Sg1 and MCF12A-Sg2) were examined by immunoblotting. **(B)** The box and whisker plot of CPT1C expression in p53 wide-type breast cancer patients (n = 657) from TCGA with high (n = 328) or low miR-200c expression (n = 329). The statistical significance of differential CPT1C expression in miR-200c high expression and low expression groups was determined by Wilcoxon rank-sum test. p: corresponding p-value. **(C, D)** Restoring the expression of miR-200c compromises the enhanced CPT1C expression caused by mutant p53. The expression of CPT1C in control (MCF12A-Ctrl), p53 mutant cells (MCF12A-p53^R249S^, MCF12A-p53^R273H^ and MCF12A-p53^R280K^) and miR-200c-overexpressing p53 mutant cells (MCF12A-p53^R249S^-miR-200c, MCF12A-p53^R273H^ -miR-200c and MCF12A-p53^R280K^-miR-200c) were examined by Q-PCR **(C)** or immunoblotting **(D)**. **(E)** The box and whisker plot of CPT1C expression in breast cancer patients (n = 290) harboring p53 missense mutations with high (n = 145) or low miR-200c expression (n = 145). Statistic method: Wilcoxon rank-sum test. p: corresponding p-value. **(F)** The box and whisker plot of CPT1C expression in total breast cancer patients (n = 1074) from TCGA with high (n = 537) or low miR-200c expression (n = 537). Statistic method: Wilcoxon rank-sum test. p: corresponding p-value. **(G)** The Spearman correlation plot for CPT1C versus miR-200c in total breast cancer patients from the TCGA database. R: correlation coefficient value; P: corresponding p-value. **(H–J)** Knockdown of ZEB2 interferes with the enhanced expression of CPT1C in miR-200c-KO **(H)**. MCF12A-miR-200c-Sg2), p53 mutant-overexpressing **(I)**, MCF12A-p53 ^R273H^), and BT549 **(J)** cells. **(K)** The Pearson’s pairwise correlation plot for CPT1C versus ZEB2 in total breast cancer patients from TCGA. R: correlation coefficient value; P: corresponding p-value. The result is derived from bc-GeneExMiner v4.5 (http://bcgenex.ico.unicancer.fr).

CPT1C is considered a direct target gene of WTp53 as WTp53 activates CPT1C expression through direct promoter binding in murine embryonic fibroblasts (13). Since p53 hot-spot mutants usually exert a dominant-negative effect by interfering with the DNA binding activity of WTp53, and the canonical p53 responsive elements were only found in the murine *Cpt1C* promoter but not in human’s (35), it is possible that Mutp53 activates CPT1C expression through an indirect mechanism in human cancer cells. In our previous study, we found that Mutp53 is able to attenuate OXPHOS through an indirect downregulation of PCK2 by disturbing the expression of miR-200c (11). In this study, the genome-wide RNA sequencing analysis in miR-200c-KO MCF12A cells revealed that miR-200c KO leads to altered expression of metabolic genes in the lipid metabolism pathway, where *CPT1C* mRNA expression is highly activated in miR-200c-KO MCF12A cells (**Supplementary Figure S6**). This elevated CPT1C expression caused by miR-200c-deficiency was further confirmed as the CPT1C protein level was substantially enhanced in miR-200c-KO MCF12A cells containing *WTp53* (MCF-12A-Sg1 and -Sg2, **Figure 6A**), and the *CPT1C* mRNA expression was higher in the cohort of p53 wild-type breast cancer patients with low expression of miR-200c (**Figure 6B**), raising the possibility that Mutp53 might activate CPT1C expression through downregulating miR-200c. To examine this hypothesis, we restored miR-200c expression in p53-R249S, p53-R273H, and p53-R280K MCF-12A cells and found that the recovery of miR-200c expression compromised both the elevated mRNA (**Figure 6C**) and protein (**Figure 6D**) expression of CPT1C caused by Mutp53, suggesting Mutp53 might upregulate CPT1C expression through interfering with the expression of miR-200c. To further confirm the antagonistic relationship between Mutp53 and miR-200c on CPT1C expression, we restored miR-200c in BT549 and 4T1 cells (11). As shown in **Supplementary Figure S7**, restoration of miR-200c led to decreased expression of CPT1C in Mutp53-harboring human BLBC cells and p53-null murine 4T1 cells. This *in vitro* observation could be recaptured in p53 mutated breast cancer patients, as the expression of CPT1C is lower in the cohort of miR-200c-High when compared with patients harboring low miR-200c expression (**Figure 6E**). Additionally, in total breast cancer patients, the miR-200c-Low cohort exhibits a higher expression of CPT1C (**Figure 6F**), and a moderate negative correlation between the expression of CPT1C and miR-200c was also revealed by Spearman correlation analysis (R=0.18, P<0.0001; **Figure 6G**). Based on these results, our study suggested that Mutp53 elevates CPT1C expression through disturbing miR-200c.

CPT1C is indirectly suppressed by miR-200c due to the lack of miR-200c seeding sequence in the 3’-UTR of CPT1C mRNA (analyzed by TargetScan, data not shown). Therefore, to investigate the molecular mechanism by which Mutp53 upregulates CPT1C through disturbing the miR-200c axis, we examined the CPT1C expression in MCF-12A-p53^R273H^, BT549, and MDA-MB-231 cells knocked down with miR-200c downstream targets including ZEB1, ZEB2, Bmi-1, or Slug, which are highly induced by miR-200c deficiency (11). As shown in **Supplementary Figure S8**, downregulating the expression of ZEB1 (**Supplementary Figure S8A**), Bmi-1 (**Supplementary Figure S8B**), or Slug (**Supplementary Figure S8C**) did not affect the expression of CPT1C in p53 mutant-expressing cells or miR-200c-deficient cells, but knockdown of ZEB2 interfered with the elevated expression of *CPT1C* caused by p53 mutation (MCF-12A-miR-200c-Sg2-siZEB2-A3, C2, D2, and E2; **Figure 6H**). Moreover, similar results were extended to ZEB2 knocked down MCF12A-p53^R273H^ (**Figure 6I**) and BT549 (**Figure 6J**) cells, and a moderate expressional correlation between CPT1C and ZEB2 was found in breast cancer patients (R=0.28; P<0.0001; **Figure 6K**). All these results together implied that Mutp53 might upregulate CPT1C through inhibiting miR-200c, subsequently compromising the suppressive effect of miR-200c on ZEB2, which in turn enhances CPT1C expression.

### High CPT1C Expression Is Associated With Poor Prognosis in Basal-Like Breast Cancer Patients

**Figure 7:**
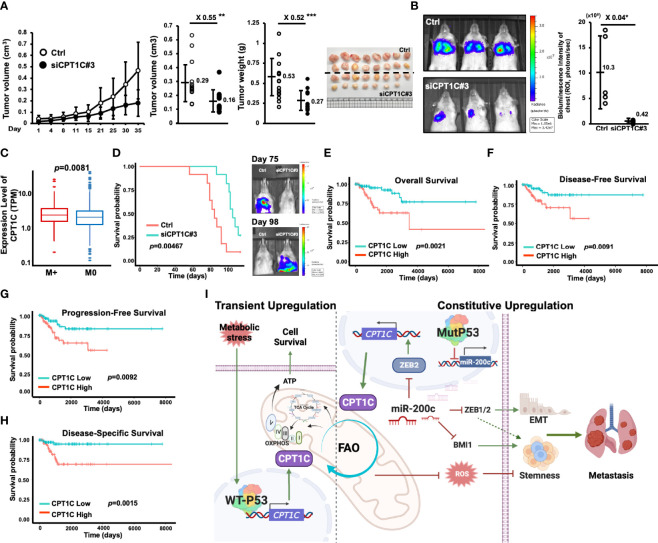
High CPT1C expression is associated with poor prognosis in basal-like breast cancer patients. **(A)** Interfering with CPT1C expression attenuates the growth of BLBCs *in vivo*. MDA-MB-231-Ctrl (n = 16) and MDA-MB-231-siCPT1C#3 (n = 15) cells (3x10^6^) were inoculated into the fourth mammary fat pad of SCID mice for five weeks. Tumor sizes were recorded at indicated days and plotted as a growth curve. At day 35, tumors were harvested surgically, tumor weights were measured, and tumor volumes were determined with the formula: (d1x d2^2^)/2 (d1: larger diameter; d2: smaller diameter) and plotted as dot plots (mean ± SD). The two sides of the lines above the dots indicated the comparison of the CPT1C-KD group to the control group, and the value of fold changes with the asterisks representing significance was marked above the line. Data were analyzed by unpaired t-test. **p < 0.01; ***p <0.001. **(B)** Interfering with CPT1C expression suppresses the pulmonary metastasis of BLBCs *in vivo.* MDA-MB-231- Luc2-Ctrl (n = 5) and MDA-MB-231-Luc2-siCPT1C#3 (n = 5) cells (1x10^6^) cells were inoculated into NOD-SCID mice through lateral tail-vein injection. At five weeks post-injection, an IVIS imaging was carried out to examine the pulmonary colonization of MDA-MB-231 cells. Left: Representative images of MDA-MB-231-Luc2 luciferase-mediated bioluminescence intensities in control and CPT1C-KD groups. Right: The total tumor growth was estimated based on luciferase activity detected as total bioluminescence intensity of Chest (photons/sec) and plotted as dot plot (mean ± SD). The two sides of the line above the dots indicated the comparison of the CPT1C-KD group to the control group, and the value of fold changes with the asterisks representing significance was marked above the line. Data were analyzed by unpaired t-test. *p <0.05. **(C)** The box and whisker plot of CPT1C expression in breast cancer patients with metastasis (M+, red, n = 184) or without metastasis (M0, blue, n = 909) from the TCGA database. The statistical significance was determined by Wilcoxon rank-sum test. p: corresponding p-value. **(D)** Low CPT1C expression confers better overall survival (OS) *in vivo*. MDA-MB-231-Ctrl (n = 11) and MDA-MB-231-siCPT1C#3 (n = 11) cells (3x10^6^) were inoculated into the fourth mammary fat pad of NOD-SCID mice. Left: The Kaplan-Meier survival plot was drawn by using IBM SPSS Statistics 24 according to the time frame from the first death to the last. The statistical significance was determined with the logrank test. Right: the represented images of IVIS analysis at day 75 and day 98 post-inoculation. At day 75, only the Ctrl group exhibited lung metastasis. No metastatic lesion was found in the CPT1C-KD group. At day 98, the only survivor (1/11) of the Ctrl group showed no sign of pulmonary metastasis. Metastatic lesions were found in all of the mice (11/11) in the CPT1C-KD group, which are dead by day 112. **(E**–**H)** High CPT1C expression predicts poor overall survival (OS; **(E)**, disease-free survival [DFS; **(F)**], progression-free survival [PFS; **(G)**], and disease-specific survival [DSS; **(H)**] in BLBC patients. The Kaplan-Meier survival estimates of differential CPT1C expression were derived from the BLBC cohort (n = 172 for OS and PFS; n = 168 for DFS; n = 154 for DSS) of TCGA RNA-Seq data. The survival curve compared the patients with High (red, n = 86 for OS and PFS; n = 84 for DFS; n = 77 for DSS) and Low (green, n = 86 for OS and PFS; n = 84 for DFS; n = 77 for DSS) expression of CPT1C. The statistical significance was determined with the logrank test. **(I)** A proposed model of mutant p53 induces FAO and stemness through upregulating CPT1C expression *via* the miR-200c-ZEB2 axis.

To validate the biological significance and examine whether our *in vitro* observation could be recaptured *in vivo*, an orthotopic xenograft tumor mouse model was used to examine the role of CPT1C in tumor growth. CPT1C-KD (MDA-MB-231-siCPT1C#3) and its control cells were inoculated into the mammary fat pad, and the tumor size was measured every three days for five weeks. As shown in **Figure 7A**, a slow tumor growth curve was observed in CPT1C-KD cells, and knockdown of CPT1C caused a near 50% decrease both in tumor volumes and tumor weights, indicating CPT1C contributes to the tumor growth of BLBC *in vivo*. Additionally, to address whether CPT1C involves in metastasis *in vivo*, CPT1C-KD cells expressing luciferase activity (MDA-MB-231-Luc2-siCPT1C#3) were inoculated through tail vein injection, and the metastatic lesions in the lung were visualized and quantified by an *in-vivo* imaging system (IVIS). As shown in **Figure 7B**, the control cells (MDA-MBA-231-Luc2-pLko.1-Ctrl) developed colonized tumor in the lung after five weeks. However, silencing CPT1C interfered with lung metastasis of BLBC cells, as the total metastatic burden quantified by luciferase activity was significantly decreased in MDA-MB-231-Luc2-siCPT1C#3 cells, indicating CPT1C might facilitate distal metastasis, which is in line with the clinical observation that CPT1C expression is higher in breast cancer patients with metastatic tumors (P<0.01; **Figure 7C**).

Furthermore, interference of CPT1C expression significantly prolonged the survival of the orthotopic tumor mouse models (**Figure 7D**, left). CPT1C-KD (MDA-MB-231-Luc2-siCPT1C#3) and the control cells (MDA-MBA-231-Luc2-pLko.1-Ctrl) were inoculated into the fourth mammary fat pads of 11 mice, respectively, in each group. The first death in control mice and CPT1C-KD mice was observed on day 56 and day 87, respectively, after inoculation, and the 50% mortality in control mice occurred within 84 days after inoculation, which was 21 days earlier than that in CPT1C-KD mice. We suggested these mice might die from metastatic burden since spontaneous metastasis from primary mammary fat pad tumors was detected by IVIS during the 112-day observation period (**Figure 7D**, right). The representative images showed that on day 75, spontaneous metastasis developed solely in control mice, which had started to die almost three weeks ago. However, on day 98, the metastatic signal was detectable on CPT1C-KD mice instead of control mice, indicating when CPT1C-KD mice began to develop metastasis, control mice had already died from metastatic disease, and those who still survived were control mice without metastatic burden. This result implied that interference of CPT1C expression hindered the spontaneous metastatic ability of BLBC cells, leading to improved disease-specific survival. The significant difference in the survival rates between control and CPT1C-KD mice not only reflected that CPT1C deficiency attenuated pulmonary colonization (**Figure 7B**), but also was in accordance with the clinical relevance of CPT1C in BLBC revealed by analyzing BLBC patients from the TCGA database. BLBC patients were divided into groups of CPT1C-High and CPT1C-Low according to the mRNA levels of *CPT1C*. As shown in **Figures 7E–H**, patients in the cohort with High CPT1C expression had much worse overall survival (OS; **Figure 7E**). Moreover, poor disease-free survival (DFS; **Figure 7F**), progression-free survival (PFS; **Figure 7G**) as well as disease-specific survival (DSS; **Figure 7H**) were observed in the CPT1C-High cohort, indicating high CPT1C expression could predict unfavorable clinical outcomes in BLBC patients, and strongly suggesting a pivotal role of CPT1C in facilitating tumor progression *in vivo*.

Since CPT1A, CPT1B, and CPT1C belong to the CPT family, we also compared the clinical relevance of CPT1A, CPT1B, and CPT1C in total breast cancer patients and patients with various subtypes of breast cancer (**Supplementary Figure S9–S12**). Differing from high expression of CPT1A that predicts a poor overall survival in total (**Supplementary Figure S9**) and luminal A (**Supplementary Figure S10**) breast cancer patients, and high expression of CPT1B that predicts a better overall survival in luminal B breast cancer patients (**Supplementary Figure S11**), high CPT1C expression specifically predicts a poor overall survival in BLBC patients, but not in total or other subtypes of breast cancers (**Supplementary Figure S12**), which further implies that targeting CPT1C, but not the other isoforms of CPT1, might be a promising treatment strategy for BLBC.

Together, the *in vivo* observation and the clinical validation revealed the critical role of CPT1C in facilitating tumor progression of BLBC and the significance of CPT1C as the prognostic indicator for patients with BLBC, reflecting our *in vitro* results that CPT1C contributes to EMT-associated phenotypes, migration, invasion, and cancer stemness in BLBC cells.

## Discussion

During the 40 years of discovery, p53 has been characterized as a pivotal tumor suppressor by ensuring genomic integrity, inducing cell-cycle arrest or apoptosis, controlling self-renewal and differentiation of stem cells, and preventing somatic cell reprogramming (36). In the past decade, p53 has emerged as an important regulator of cellular metabolism, in which perturbations of p53-associated metabolic networks could lead to the burden of cancers (37). Our previous study reveals that dysfunction of p53 attenuates OXPHOS by downregulating the miR-200c-PCK2 axis in BLBC (11), providing robust evidence for the causal link between Mutp53-mediated metabolic reprogramming and cancer stemness. Despite this, the complexity of p53 pathways and the cell type-specific regulation of metabolism still limit our understanding of the connection between cancer-associated metabolic rewiring and p53 deficiency-induced biological functions. Therefore, figuring out the complicated p53 networks of metabolic regulation and the underlying molecular mechanisms would be a major breakthrough in cancer metabolism, which paves the way for the development of precise metabolic therapies for specific cancers.

In response to stress conditions, such as oxidative stress and hypoxia, p53 is activated to modulate its target genes involved in DNA repair and cell survival (38). WTp53 has been reported to induce CPT1C expression under hypoxia and glucose deprivation, which promotes tumor cell survival (13). In addition, CPT1C promotes ATP production from FAO to protect tumor cells from metabolic stress (30). Although p53 directly transactivates *Cpt1c* in murine cells (13), whether *CPT1C* is a direct target gene of p53 in human is still under debate since the lack of direct interaction between WTp53 and the *CPT1C* promoter revealed by ChIP assay is reported by a recent study (35). In our study, we found that Mutp53 enhances FAO to facilitate the progression of BLBC by upregulating CPT1C through the miR-200c-ZEB2 axis. The FAO activity was measured by using Mito Stress assay with palmitate as substrates and presented as a basal ratio, indicating the portion of FAO-contributed OCR to the total cellular oxygen consumption (**Figures 1B–F**). As shown in **Figure 1G**, overexpressing Mutp53 in normal mammary epithelial cells led to significant increases in the basal ratio, implying Mutp53 enhances FAO activity. However, the treatment of FCCP, a mitochondrial uncoupler disrupting the proton gradient across the mitochondrial inner membrane and leading to the maximal OCR (39), resulted in a decreased OCR instead of an increased OCR in Mutp53-bearing cells (**Figures 1C–F**). The reason for this paradox might be the concentration of FCCP used in our FAO assay was the optimized concentration for that specific cell line in the Mito Stress assay, in which cells are maintained in the medium containing glucose. In contrast, in the FAO assay, cells were starved in the glucose-free medium containing fatty acids with even micromolar concentrations of ETO that could inhibit FCCP-stimulated oxygen consumption (40). Additionally, high concentrations of FCCP can be inhibitory to the OCR rather than stimulatory due to the damaged mitochondrial and loss of membrane potential caused by the toxicity (41–43). One of the possible solutions to this issue is to perform a titration of FCCP to optimize the appropriate concentrations under the conditions of the FAO assay; though, it is not the priority in our current study because the basal ratio we used to represent the FAO activity depends on the basal OCR but not the maximal respiration or the spare respiratory capacity determined by FCCP. Therefore, the optimized concentration of FCCP for cells used in the FAO assay would be further determined in the future when only necessary.

Another important and novel observation in our present work is that Mutp53 promotes FAO activity through activating CPT1C, which seems contradictory to the current general concept that WTp53 promotes FAO *via* inducing CPT1C. In specific circumstances, Mutp53 would induce the expression of WTp53 target genes. For example, Tran et al. demonstrate that Mutp53 hyper-transactivates target genes of WTp53, including *CDKN1A*, *TIGAR*, *GLS2*, and *GADD45A*, to protect cancer cells against glutamine deprivation (44). WTp53 is activated upon acute stress stimuli, but for tumor cells confronted with various types of stress, especially the severe oxidative stress during metastasis (45), continuous activation of WTp53 is deleterious. Due to the extended half-life and thus accumulation of Mutp53 in tumors (46), we suggest that Mutp53 provides sustained beneficial effects for tumor cells than transient effects induced by WTp53. Therefore, BLBC cells are able to maintain a constitutive expression of CPT1C by Mutp53, which is critical for BLBC progression (**Figure 7I**).

During the loss of attachment to the extracellular matrix, which is the prerequisite of metastasis (47), tumor cells display inhibited glucose uptake, reduced ATP production as well as increased ROS, followed by induction of anoikis, a form of apoptosis due to loss of cell-matrix interactions (33, 48). Several lines of evidence reveal that FAO is critical for preventing detached cells from undergoing anoikis by providing extra ATP and maintaining redox homeostasis in breast cancers (49, 50), which is in line with our observations that both etomoxir treatment and CPT1C deficiency impaired migration, invasion, mammosphere formation, and anchorage-independent colony formation. Therefore, our results indicate that accumulated Mutp53 proteins lead to prolonged induction of CPT1C, potentiating FAO, which contributes to increased cellular motility and stemness in BLBC cells. CPT1C has been found by others to facilitate cancer progression by promoting proliferation, tumor growth, and survival, increasing ATP synthesis to protect against metabolic stress, contributing to chemoresistance (51), and preventing cancer cell senescence (52). To our knowledge, we first uncovered the novel roles of CPT1C in regulating EMT, cellular motility, cancer stemness, and metastasis. Furthermore, the extremely high relevance of CPT1C with clinical parameters implies that CPT1C is highly associated with tumor progression in BLBC, which is in accordance with our *in vitro* results and *in vivo* tumorigenic and metastatic mouse models.

FAO produces NADH, FADH2, and acetyl-CoA, which fuels OXPHOS directly or through the TCA cycle, hence cellular factors or signaling events could potentiate OXPHOS activity by enhancing FAO (17, 53, 54). Although OXPHOS plays a key role in regulating cellular redox status, the FAO-enhanced OXPHOS activity might not increase oxidative stress accordingly. A study by Choi et al. reveals that in spite of the increased OXPHOS in drug-resistant gastric CSCs, NADPH regeneration fueled by the enhanced FAO activity lowers the ROS level. In contrast, inhibition of FAO by ETO reduces the NADPH pool and increases mitochondrial ROS levels in CSCs (55). FAO is one of the major sources of cytosolic NADPH, which is utilized by a wide variety of cancer cells to overcome oxidative stress (33, 56). For example, inhibiting FAO by ETO decreases NADPH production, increases ROS level, leading to oxidative stress, ATP depletion, and cell death in glioblastoma cells (57). Furthermore, CPT has been reported to contribute to the generation of NADPH in several different cancer types. Wang et al. demonstrate that CPT1A-induced FAO and CPT1A-maintained NADPH/NADP^+^ ratio are critical for redox homeostasis of detached CRC cells, in which NAC treatment increased anchorage-independent growth of detached CPT1A-KD CRC cells (34). In another study, PGC-1α, the key transcription coactivator regulating CPT1A and CPT1B, binds to the transcription factor CEBPB to promote CPT1A expression, which enhances FAO activity to maintain the high NADPH/NADP^+^ ratio, contributing to radiation resistance of nasopharyngeal carcinoma cells (58). In addition to CPT1, elevated expression of CPT2 in gastrointestinal cancer cells is also important for fueling the NADPH pool to maintain redox homeostasis upon chemo-drug treatment (59). Together, all of these findings are consistent with ours as a higher NADPH/NADP^+^ ratio was observed in p53-R280K MCF12A cells (**Figure 5G**), and knockdown of CPT1C in p53-R273H MCF12A cells and MDA-MB-231 cells increased oxidative stress as measured by CellROX™ Green Reagent (**Figures 5A, C**) and DCFDA (**Figures 5B, D**). Moreover, CPT1C deficiency-induced oxidative stress could be rescued by NAC treatment (**Figures 5A–D**), indicating that CPT1C-driven FAO increases the generation of NADPH, which is utilized by BLBC cells to reduce oxidative stress, maintain an optimized redox equilibrium, and facilitate migration and stemness.

Paradoxically, a high NADPH/NADP^+^ ratio can play a double-faceted role in regulating redox status by promoting both antioxidant and prooxidative pathways. As an antioxidant, NADPH is used by glutathione reductase to generate glutathione (GSH), which controls and maintains redox status by scavenging several ROS (60). However, GSH not only eliminates ROS, but also protects against nitrosative stress by buffering nitric oxide (NO), which can react with and damage cellular macromolecules like DNA, proteins, and lipids (61). Therefore, GSH is a critical antioxidant controlling cellular redox homeostasis by neutralizing ROS and reactive nitrogen species (RNS). It is noteworthy that the DCFDA fluorogenic probe used in our study is oxidized not only by H_2_O_2_ but also by RNS (62, 63); hence, CPT1C-mediated control on the redox status might be through increasing GSH level by enhancing FAO to fuel the NADPH pool, which then utilized by BLBC cells to neutralize both ROS and RNS. The decreased oxidative stress was further confirmed by using another non-specific fluorogenic probe, CellROX™ Green Reagent, which is commonly used to compare total oxidative stress status.

In addition to reducing oxidative stress, CPT1C-mediated induction of EMT and enhanced stemness may be achieved by epigenetic modification since lipid serves as a major source for histone acetylation to activate gene expression (64). Whether CPT1C exerts its oncogenic functions through epigenetic control by increasing the pool of acetyl-coA from FAO for histone acetylation to activate genes involved in EMT and stemness needs further study.

Taken together, the striking consistency among our *in vitro* and *in vivo* results, as well as clinical validation strongly supports our model that Mutp53 potentiates FAO through constitutively upregulating CPT1C *via* dysregulating the miR-200c-ZEB2 axis, which in turn facilitates EMT-associated phenotypes, enhances cancer stemness, and promotes metastasis through modulating cellular redox status, leading to the progression of BLBC and the poor clinical outcomes in BLBC patients (**Figure 7I**). Our current study fully addresses the indispensable connections between Mutp53-driven metabolic reprogramming and the highly malignant characteristics of BLBC, providing a reasonable link between the extremely high frequency of p53 mutation and the reliance on FAO in BLBC. It is noteworthy that since the mediator of Mutp53-induced oncogenic events, CPT1C, is brain-specific, existing only in neurons while is overexpressed in a wide range of cancers (51), and a vast majority of small molecule drugs do not cross the blood-brain barrier (65), it is really promising to develop CPT1C inhibitors with maximal efficacy and minimal off-target effects. In conclusion, our present work unravels the previously unconfirmed function of Mutp53 in FAO, depicts the detailed molecular mechanism accounting for Mutp53-mediated metabotypes, and uncovers the novel biological roles of CPT1C in cancer progression, which is not only a milestone for the thorough understanding of the p53 regulatory network of metabolism but also an indication that targeting Mutp53-miR-200c-ZEB2-CPT1C axis might be developed into a novel and effective therapies for BLBC in the imminent future.

## Data Availability Statement

The original contributions presented in the study are included in the article/**Supplementary Materials**. Further inquiries can be directed to the corresponding author.

## Ethics Statement

The animal study was reviewed and approved by Institutional Animal Care and Use Committee at National Yang Ming Chiao Tung University, Taiwan. (IACUC number: NCTU-IACUC-106030).

## Author Contributions

C-YW: Investigation, data curation, writing-original draft, writing-review and editing. C-HW: Investigation, data curation, methodology, visualization. R-TM: Funding acquisition, investigation, data curation, methodology. T-WC: Funding acquisition, visualization, data curation, investigation. C-WL: Funding acquisition, methodology, writing-review and editing. C-HC:Conceptualization, data curation, investigation, methodology, funding acquisition, project administration, visualization, writing-original draft, writing-review and editing. All authors contributed to the article and approved the submitted version.

## Funding

This work was financially supported in part by the following: Ministry of Science and Technology (105-2320-B-009-004, 106-2320-B-009-002, 107-2628-B-009-002, 108-2628-B-009-002 and 109-2628-B-009-004 to Dr. C.-H. Chao; 109-2311-B-009-002 and 110-2311-B-A49-001 to Dr. T.-W. Chen; 109-2314-B-001-002 and 109-2314-B-001-008 to Dr. C.-W. Li; 107-2320-B-009-006-MY2 and 109-2320-B-009-002 to Dr. R.-T. Mai). The “Smart Platform of Dynamic Systems Biology for Therapeutic Development” and “Center for Intelligent Drug Systems and Smart Bio-devices (IDS^2^B) from The Featured Areas Research Center Program” within the framework of the Higher Education Sprout Project of the Ministry of Education (MOE) in Taiwan. We would also like to thank the National Core Facility for Biopharmaceuticals (NCFB, MOST 106-2319-B-492-002) and the National Center for High-performance Computing (NCHC) of National Applied Research Laboratories (NARLabs) of Taiwan for providing computational resources and storage resources, and the National RNAi Core Facility at Academia Sinica in Taiwan for providing shRNA constructs.

## Conflict of Interest

The authors declare that the research was conducted in the absence of any commercial or financial relationships that could be construed as a potential conflict of interest.

## Publisher’s Note

All claims expressed in this article are solely those of the authors and do not necessarily represent those of their affiliated organizations, or those of the publisher, the editors and the reviewers. Any product that may be evaluated in this article, or claim that may be made by its manufacturer, is not guaranteed or endorsed by the publisher.

## References

[1] Vogelstein B Sur S Prives C. P53: The Most Frequently Altered Gene in Human Cancers. Nat Educ (2010) 3(9):6.

[2] Leroy B Anderson M Soussi T. TP53 Mutations in Human Cancer: Database Reassessment and Prospects for the Next Decade. Hum Mutat (2014) 35(6):672–88. doi: 10.1002/humu.22552 24665023

[3] Olivier M Hollstein M Hainaut P. TP53 Mutations in Human Cancers: Origins, Consequences, and Clinical Use. Cold Spring Harb Perspect Biol (2010) 2(1):a001008. doi: 10.1101/cshperspect.a001008 20182602 PMC2827900

[4] Joerger A Fersht A. Structure–Function–Rescue: The Diverse Nature of Common P53 Cancer Mutants. Oncogene (2007) 26(15):2226–42. doi: 10.1038/sj.onc.1210291 17401432

[5] Brosh R Rotter V. When Mutants Gain New Powers: News From the Mutant P53 Field. Nat Rev Cancer (2009) 9(10):701–13. doi: 10.1038/nrc2693 19693097

[6] Anders CK Carey LA. Biology, Metastatic Patterns, and Treatment of Patients With Triple-Negative Breast Cancer. Clin Breast Cancer (2009) 9 Suppl 2:S73–81. doi: 10.3816/CBC.2009.s.008 PMC2919761 19596646

[7] Yadav BS Chanana P Jhamb S. Biomarkers in Triple Negative Breast Cancer: A Review. World J Clin Oncol (2015) 6(6):252–63. doi: 10.5306/wjco.v6.i6.252 PMC4675910 26677438

[8] Liu J Zhang C Hu W Feng Z. Tumor Suppressor P53 and Metabolism. J Mol Cell Biol (2019) 11(4):284–92. doi: 10.1093/jmcb/mjy070 PMC6487777 30500901

[9] Zhang C Liu J Liang Y Wu R Zhao Y Hong X. Tumour-Associated Mutant P53 Drives the Warburg Effect. Nat Commun (2013) 4(1):1–15. doi: 10.1038/ncomms3935 PMC3969270 24343302

[10] Kim H-R Roe J-S Lee J-E Cho E-J Youn H-D. P53 Regulates Glucose Metabolism by miR-34a. Biochem Biophysi Res Commun (2013) 437(2):225–31. doi: 10.1016/j.bbrc.2013.06.043 23796712

[11] Chao C-H Wang C-Y Wang C-H Chen T-W Hsu H-Y Huang H-W. Mutant P53 Attenuates Oxidative Phosphorylation and Facilitates Cancer Stemness Through Downregulating miR-200c-PCK2 Axis in Basal-Like Breast Cancer. Mol Cancer Res (2021) 19(11):1900–16. doi: 10.1158/1541-7786.MCR-21-0098 34312289

[12] Parrales A Iwakuma T. P53 as a Regulator of Lipid Metabolism in Cancer. Int J Mol Sci (2016) 17(12):2074. doi: 10.3390/ijms17122074 PMC5187874 27973397

[13] Sanchez-Macedo N Feng J Faubert B Chang N Elia A Rushing E. Depletion of the Novel P53-Target Gene Carnitine Palmitoyltransferase 1c Delays Tumor Growth in the Neurofibromatosis Type I Tumor Model. Cell Death Differ (2013) 20(4):659–68. doi: 10.1038/cdd.2012.168 PMC3595492 23412344

[14] Camarda R Zhou AY Kohnz RA Balakrishnan S Mahieu C Anderton B. Inhibition of Fatty Acid Oxidation as a Therapy for Myc-Overexpressing Triple-Negative Breast Cancer. Nat Med (2016) 22(4):427–32. doi: 10.1038/nm.4055 PMC4892846 26950360

[15] Zhang C Yue C Herrmann A Song J Egelston C Wang T. STAT3 Activation-Induced Fatty Acid Oxidation in Cd8+ T Effector Cells Is Critical for Obesity-Promoted Breast Tumor Growth. Cell Metab (2020) 31(1):148–61. e5. doi: 10.1016/j.cmet.2019.10.013 31761565 PMC6949402

[16] Park JH Vithayathil S Kumar S Sung P-L Dobrolecki LE Putluri V. Fatty Acid Oxidation-Driven Src Links Mitochondrial Energy Reprogramming and Oncogenic Properties in Triple-Negative Breast Cancer. Cell Rep (2016) 14(9):2154–65. doi: 10.1016/j.celrep.2016.02.004 PMC4809061 26923594

[17] Wright HJ Hou J Xu B Cortez M Potma EO Tromberg BJ. CDCP1 Drives Triple-Negative Breast Cancer Metastasis Through Reduction of Lipid-Droplet Abundance and Stimulation of Fatty Acid Oxidation. Proc Natl Acad Sci (2017) 114(32):E6556–65. doi: 10.1073/pnas.1703791114 PMC5559020 28739932

[18] van Weverwijk A Koundouros N Iravani M Ashenden M Gao Q Poulogiannis G. Metabolic Adaptability in Metastatic Breast Cancer by AKR1B10-Dependent Balancing of Glycolysis and Fatty Acid Oxidation. Nat Commun (2019) 10(1):1–13. doi: 10.1038/s41467-019-10592-4 31221959 PMC6586667

[19] Wang T Fahrmann JF Lee H Li Y-J Tripathi SC Yue C. JAK/STAT3-Regulated Fatty Acid B-Oxidation Is Critical for Breast Cancer Stem Cell Self-Renewal and Chemoresistance. Cell Metab (2018) 27(1):136–50.e5. doi: 10.1016/j.cmet.2017.11.001 29249690 PMC5777338

[20] Havas KM Milchevskaya V Radic K Alladin A Kafkia E Garcia M. Metabolic Shifts in Residual Breast Cancer Drive Tumor Recurrence. J Clin Investig (2017) 127(6):2091–105. doi: 10.1172/JCI89914 PMC5451224 28504653

[21] Chang C-J Chao C-H Xia W Yang J-Y Xiong Y Li C-W. P53 Regulates Epithelial–Mesenchymal Transition and Stem Cell Properties Through Modulating Mirnas. Nat Cell Biol (2011) 13(3):317–23. doi: 10.1038/ncb2173 PMC3075845 21336307

[22] Li B Dewey CN. Rsem: Accurate Transcript Quantification From RNA-Seq Data With or Without a Reference Genome. BMC Bioinf (2011) 12(1):1–16. doi: 10.1186/1471-2105-12-323 PMC3163565 21816040

[23] Parker JS Mullins M Cheang MC Leung S Voduc D Vickery T. Supervised Risk Predictor of Breast Cancer Based on Intrinsic Subtypes. J Clin Oncol (2009) 27:1160–7. doi: 10.1200/JCO.2008.18.1370 PMC2667820 19204204

[24] Koboldt D Fulton R McLellan M Schmidt H Kalicki-Veizer J McMichael J. Comprehensive Molecular Portraits of Human Breast Tumours. Nature (2012) 490(7418):61–70. doi: 10.1038/nature11412 23000897 PMC3465532

[25] Li Q Wang K. Intervar: Clinical Interpretation of Genetic Variants by the 2015 Acmg-Amp Guidelines. Am J Hum Genet (2017) 100(2):267–80. doi: 10.1016/j.ajhg.2017.01.004 PMC5294755 28132688

[26] Liu J Lichtenberg T Hoadley KA Poisson LM Lazar AJ Cherniack AD. An Integrated TCGA Pan-Cancer Clinical Data Resource to Drive High-Quality Survival Outcome Analytics. Cell (2018) 173(2):400–16. doi: 10.1016/j.cell.2018.02.052 PMC6066282 29625055

[27] Wang D Green MF McDonnell E Hirschey MD. Oxygen Flux Analysis to Understand the Biological Function of Sirtuins. Methods Mol Biol (2013) 1077:241–58. doi: 10.1007/978-1-62703-637-5_16 PMC3817486 24014411

[28] Alvarado-Ortiz E de la Cruz-López K Becerril-Rico J Sarabia-Sánchez M Ortiz-Sánchez E García-Carrancá A. Mutant P53 Gain-Of-Function: Role in Cancer Development, Progression, and Therapeutic Approaches. Front Cell Dev Biol (2020) 8:607670–0. doi: 10.3389/fcell.2020.607670 PMC7905058 33644030

[29] Sierra AY Gratacós E Carrasco P Clotet J Ureña J Serra D. CPT1C Is Localized in Endoplasmic Reticulum of Neurons and Has Carnitine Palmitoyltransferase Activity. J Biol Chem (2008) 283(11):6878–85. doi: 10.1074/jbc.M707965200 18192268

[30] Zaugg K Yao Y Reilly PT Kannan K Kiarash R Mason J. Carnitine Palmitoyltransferase 1c Promotes Cell Survival and Tumor Growth Under Conditions of Metabolic Stress. Genes Dev (2011) 25(10):1041–51. doi: 10.1101/gad.1987211 PMC3093120 21576264

[31] Wolfgang MJ Kurama T Dai Y Suwa A Asaumi M Matsumoto S-I. The Brain-Specific Carnitine Palmitoyltransferase-1c Regulates Energy Homeostasis. Proc Natl Acad Sci (2006) 103(19):7282–7. doi: 10.1073/pnas.0602205103 PMC1564279 16651524

[32] Ma Y Temkin SM Hawkridge AM Guo C Wang W Wang X-Y. Fatty Acid Oxidation: An Emerging Facet of Metabolic Transformation in Cancer. Cancer Lett (2018) 435:92–100. doi: 10.1016/j.canlet.2018.08.006 30102953 PMC6240910

[33] Carracedo A Cantley LC Pandolfi PP. Cancer Metabolism: Fatty Acid Oxidation in the Limelight. Nat Rev Cancer (2013) 13(4):227–32. doi: 10.1038/nrc3483 PMC3766957 23446547

[34] Wang Y-N Zeng Z-L Lu J Wang Y Liu Z-X He M-M. CPT1A-Mediated Fatty Acid Oxidation Promotes Colorectal Cancer Cell Metastasis by Inhibiting Anoikis. Oncogene (2018) 37(46):6025–40. doi: 10.1038/s41388-018-0384-z 29995871

[35] Fischer M. Conservation and Divergence of the P53 Gene Regulatory Network Between Mice and Humans. Oncogene (2019) 38(21):4095–109. doi: 10.1038/s41388-019-0706-9 PMC6755996 30710145

[36] Boutelle AM Attardi LD. P53 and Tumor Suppression: It Takes a Network. Trends Cell Biol (2021) 31(4):298–310. doi: 10.1016/j.tcb.2020.12.011 33518400 PMC7954925

[37] Lahalle A Lacroix M De Blasio C Cissé MY Linares LK Le Cam L. The P53 Pathway and Metabolism: The Tree That Hides the Forest. Cancers (2021) 13(1):133. doi: 10.3390/cancers13010133 PMC7796211 33406607

[38] Joerger AC Fersht AR. The P53 Pathway: Origins, Inactivation in Cancer, and Emerging Therapeutic Approaches. Annu Rev Biochem (2016) 85:375–404. doi: 10.1146/annurev-biochem-060815-014710 27145840

[39] Muller B Lewis N Adeniyi T Leese HJ Brison DR Sturmey RG. Application of Extracellular Flux Analysis for Determining Mitochondrial Function in Mammalian Oocytes and Early Embryos. Sci Rep (2019) 9(1):1–14. doi: 10.1038/s41598-019-53066-9 31727902 PMC6856134

[40] Divakaruni AS Hsieh WY Minarrieta L Duong TN Kim KK Desousa BR. Etomoxir Inhibits Macrophage Polarization by Disrupting CoA Homeostasis. Cell Metab (2018) 28(3):490–503.e7. doi: 10.1016/j.cmet.2018.06.001 30043752 PMC6125190

[41] Dranka BP Benavides GA Diers AR Giordano S Zelickson BR Reily C. Assessing Bioenergetic Function in Response to Oxidative Stress by Metabolic Profiling. Free Radic Biol Med (2011) 51(9):1621–35. doi: 10.1016/j.freeradbiomed.2011.08.005 PMC3548422 21872656

[42] Hill BG Benavides GA Lancaster JR Ballinger S Dell’Italia L Zhang J. Integration of Cellular Bioenergetics With Mitochondrial Quality Control and Autophagy. Biol Chem (2012) 393(12):1485–512. doi: 10.1515/hsz-2012-0198 PMC3594552 23092819

[43] Abe Y Sakairi T Kajiyama H Shrivastav S Beeson C Kopp JB. Bioenergetic Characterization of Mouse Podocytes. Am J Physiol Cell Physiol (2010) 299(2):C464–C76. doi: 10.1152/ajpcell.00563.2009 PMC2928644 20445170

[44] Tran TQ Lowman XH Reid MA Mendez-Dorantes C Pan M Yang Y. Tumor-Associated Mutant P53 Promotes Cancer Cell Survival Upon Glutamine Deprivation Through P21 Induction. Oncogene (2017) 36(14):1991–2001. doi: 10.1038/onc.2016.360 27721412 PMC5383530

[45] Hayes JD Dinkova-Kostova AT Tew KD. Oxidative Stress in Cancer. Cancer Cell (2020) 38(2):167–97. doi: 10.1016/j.ccell.2020.06.001 PMC7439808 32649885

[46] Bullock AN Fersht AR. Rescuing the Function of Mutant P53. Nat Rev Cancer (2001) 1(1):68–76. doi: 10.1038/35094077 11900253

[47] Fidler IJ. The Pathogenesis of Cancer Metastasis: The 'Seed and Soil' Hypothesis Revisited. Nat Rev Cancer (2003) 3(6):453–8. doi: 10.1038/nrc1098 12778135

[48] Frisch SM Francis H. Disruption of Epithelial Cell-Matrix Interactions Induces Apoptosis. J Cell Biol (1994) 124(4):619–26. doi: 10.1083/jcb.124.4.619 PMC2119917 8106557

[49] Schafer ZT Grassian AR Song L Jiang Z Gerhart-Hines Z Irie HY. Antioxidant and Oncogene Rescue of Metabolic Defects Caused by Loss of Matrix Attachment. Nature (2009) 461(7260):109–13. doi: 10.1038/nature08268 PMC2931797 19693011

[50] Carracedo A Weiss D Leliaert AK Bhasin M De Boer VC Laurent G. A Metabolic Prosurvival Role for Pml in Breast Cancer. J Clin Investig (2012) 122(9):3088–100. doi: 10.1172/JCI62129 PMC3433768 22886304

[51] Reilly PT Mak TW. Molecular Pathways: Tumor Cells Co-Opt the Brain-Specific Metabolism Gene CPT1C to Promote Survival. Clin Cancer Res (2012) 18(21):5850–5. doi: 10.1158/1078-0432.CCR-11-3281 22952346

[52] Wang Y Chen Y Guan L Zhang H Huang Y Johnson CH. Carnitine Palmitoyltransferase 1c Regulates Cancer Cell Senescence Through Mitochondria-Associated Metabolic Reprograming. Cell Death Differ (2018) 25(4):735–48. doi: 10.1038/s41418-017-0013-3 PMC5864250 29317762

[53] Pompura SL Wagner A Kitz A LaPerche J Yosef N Dominguez-Villar M. Oleic Acid Restores Suppressive Defects in Tissue-Resident FOXP3 Tregs From Patients With Multiple Sclerosis. J Clin Investig (2021) 131(2), 1–15. doi: 10.1172/JCI138519 PMC7810477 33170805

[54] Wu D Sanin DE Everts B Chen Q Qiu J Buck MD. Type 1 Interferons Induce Changes in Core Metabolism That Are Critical for Immune Function. Immunity (2016) 44(6):1325–36. doi: 10.1016/j.immuni.2016.06.006 PMC5695232 27332732

[55] Choi H-J Jhe Y-L Kim J Lim JY Lee JE Shin M-K. FOXM1-Dependent and Fatty Acid Oxidation-Mediated ROS Modulation Is a Cell-Intrinsic Drug Resistance Mechanism in Cancer Stem-Like Cells. Redox Biol (2020) 36:101589. doi: 10.1016/j.redox.2020.101589 32521504 PMC7286985

[56] Ju H-Q Lin J-F Tian T Xie D Xu R-H. NADPH Homeostasis in Cancer: Functions, Mechanisms and Therapeutic Implications. Signal Transduct Target Ther (2020) 5(1):1–12. doi: 10.1038/s41392-020-00326-0 33028807 PMC7542157

[57] Pike LS Smift AL Croteau NJ Ferrick DA Wu M. Inhibition of Fatty Acid Oxidation by Etomoxir Impairs NADPH Production and Increases Reactive Oxygen Species Resulting in ATP Depletion and Cell Death in Human Glioblastoma Cells. Biochim Biophys Acta Bioenerg (2011) 1807(6):726–34. doi: 10.1016/j.bbabio.2010.10.022 21692241

[58] Du Q Tan Z Shi F Tang M Xie L Zhao L. PGC-1α/CEBPB/CPT1A Axis Promotes Radiation Resistance of Nasopharyngeal Carcinoma Through Activating Fatty Acid Oxidation. Cancer Sci (2019) 110(6):2050–62. doi: 10.1111/cas.14011 PMC6550130 30945396

[59] Wang Y Lu J-H Wang F Wang Y-N He M-M Wu Q-N. Inhibition of Fatty Acid Catabolism Augments the Efficacy of Oxaliplatin-Based Chemotherapy in Gastrointestinal Cancers. Cancer Lett (2020) 473:74–89. doi: 10.1016/j.canlet.2019.12.036 31904482

[60] Baldelli S Ciccarone F Limongi D Checconi P Palamara AT Ciriolo MR. Glutathione and Nitric Oxide: Key Team Players in Use and Disuse of Skeletal Muscle. Nutrients (2019) 11(10):2318. doi: 10.3390/nu11102318 PMC6836164 31575008

[61] Aquilano K Baldelli S Ciriolo MR. Glutathione: New Roles in Redox Signaling for an Old Antioxidant. Front Pharmacol (2014) 5:196. doi: 10.3389/fphar.2014.00196 25206336 PMC4144092

[62] Kalyanaraman B Darley-Usmar V Davies KJ Dennery PA Forman HJ Grisham MB. Measuring Reactive Oxygen and Nitrogen Species With Fluorescent Probes: Challenges and Limitations. Free Radic Biol Med (2012) 52(1):1–6. doi: 10.1016/j.freeradbiomed.2011.09.030 22027063 PMC3911769

[63] Eruslanov E Kusmartsev S. Identification of ROS Using Oxidized DCFDA and Flow-Cytometry. Advanced Protoc Oxid Stress Ii (2010), 594, 57–72. doi: 10.1007/978-1-60761-411-1_4 20072909

[64] McDonnell E Crown SB Fox DB Kitir B Ilkayeva OR Olsen CA. Lipids Reprogram Metabolism to Become a Major Carbon Source for Histone Acetylation. Cell Rep (2016) 17(6):1463–72. doi: 10.1016/j.celrep.2016.10.012 PMC5123807 27806287

[65] Pardridge W. Drug Targeting, Drug Discovery, and Brain Drug Development. Brain Drug Target: Future Brain Drug Dev (2001) 1:1–12. doi: 10.1017/CBO9780511549571.002

