# Supplementary material for: Mutant p53-microRNA-200c-ZEB2-Axis-Induced CPT1C Elevation Contributes to Metabolic Reprogramming and Tumor Progression in Basal-Like Breast Cancers
Source: Front Oncol. 2022 Jul 21;12:940402. doi: 10.3389/fonc.2022.940402 (PMC9351713; doi:10.3389/fonc.2022.940402)
Supplement: Supplementary file 1 [file DataSheet_1.pdf]

Suppl. Fig. S1

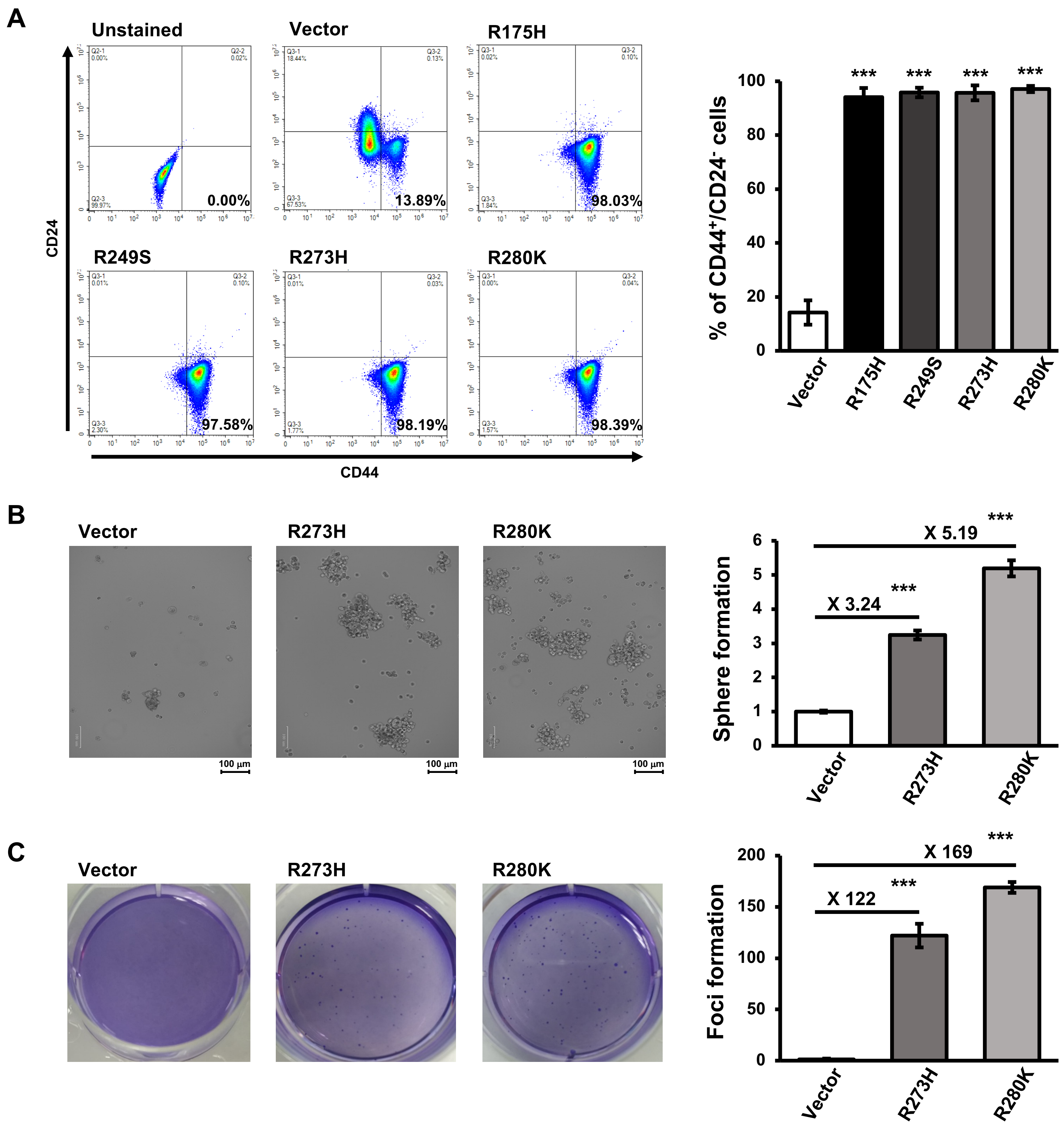
A
Unstained
Vector
R175H
R249S
R273H
R280K
CD24
CD44
0.00%
13.89%
98.03%
97.58%
98.19%
98.39%
% of CD44+/CD24- cells
***
Vector
R175H
R249S
R273H
R280K
B
Vector
R273H
R280K
100 μm
Sphere formation
X 3.24
X 5.19
***
C
Vector
R273H
R280K
Foci formation
X 122
X 169
***

Supplementary Figure S1. Mutant p53-induced biological effects in mammary epithelial cells.
A and **B**, Mutant p53 enhances stemness. **A**, left: The stem cell-enriched population in p53 mutant MCF12A cells was analyzed by FACS analysis using $CD44^+/CD24^-$ as stem cell markers. Right: Quantification of the percentage of $CD44^+/CD24^-$ population, Data shown was derived from three independent experiments (mean $\pm$ SD; n=3). **B**, p53 mutant MCF12A cells exhibit higher mammosphere forming ability. $1x10^5$ cells were grown in suspension culture for 7-8 days to form mammospheres. Mammospheres with a diameter larger than 40 μm were counted and presented as Sphere formation. Results were derived from experiments done in triplicate (mean $\pm$ SD; n=3). Representative micrographs of mammospheres under a microscope are shown. **C**, p53 mutation enhances anchorage-independent growth of mammary epithelial cells. Soft agar foci formation assay was performed to determine the anchorage-independent growth ability of p53 mutant mammary epithelial cells. Foci number in triplicate dishes was plotted as mean $\pm$ SD. The data shown is a representative of three independent experiments. Representative micrographs of stained colonies in the dishes are shown. Data were analyzed by unpaired t-test, and a p-value < 0.05 was considered statistically significant. *p <0.05, **p <0.01, ***p <0.001.

Suppl. Fig. S2

Wang *et al.*

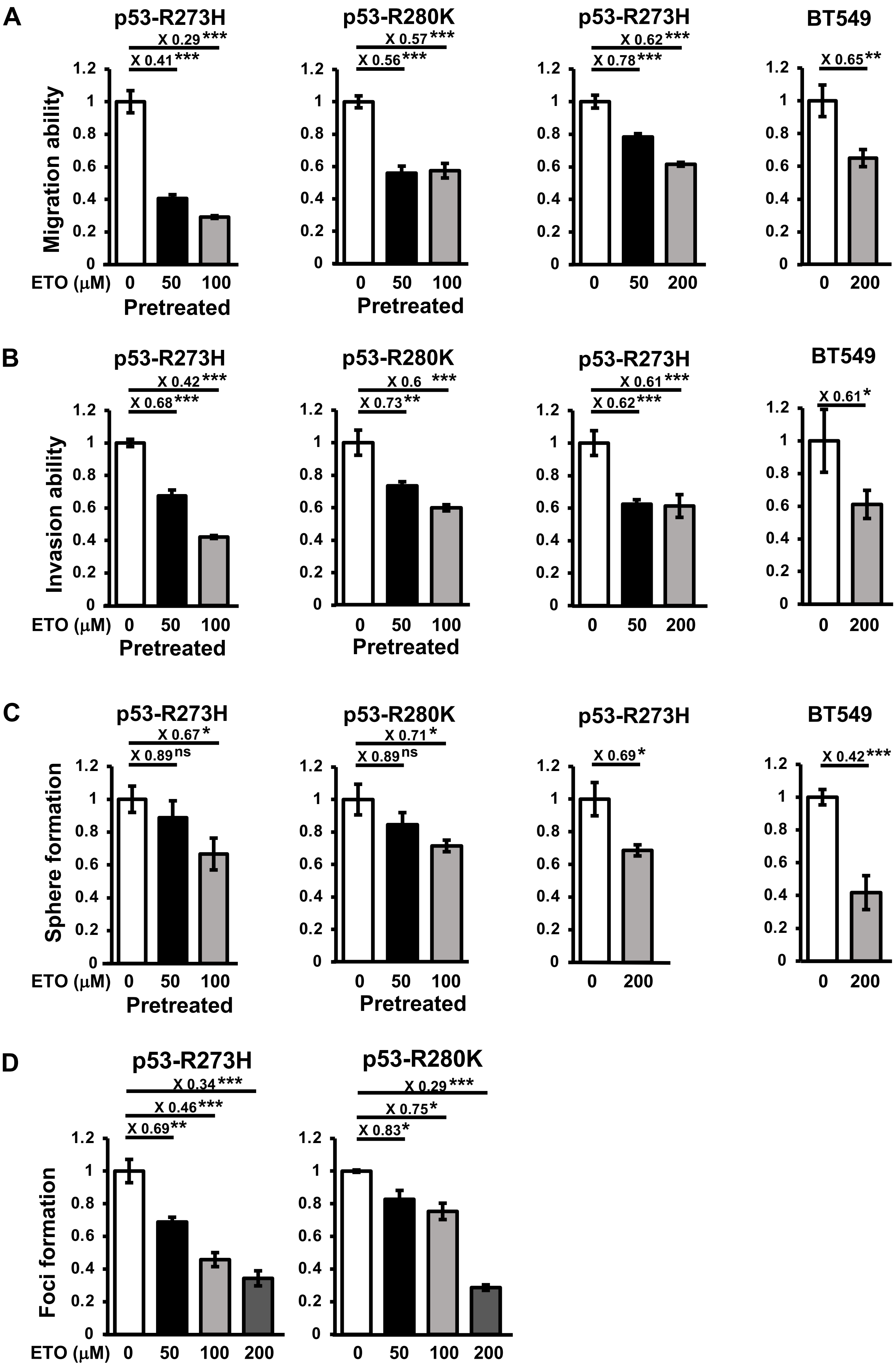

Suppl. Fig. S2 (cont.)

Supplementary Figure S2. Etomoxir treatment interferes with Mutp53-induced biological effects. **A-C**, etomoxir treatment interferes with Mutp53-inudced migration (**A**), invasion (**B**) and sphere forming (**C**) ability. Left: MCF12A-p53^{R273H} and MCF12A-p53^{R280K} cells were pretreated with etomoxir (ETO, 0, 50 or 100 μM) for 7 days then subjected to migration, invasion and sphere formation assay in the absence of continuous ETO treatment. Right: MCF12A-p53^{R273H} and BT549 cells were subjected to migration, invasion and sphere formation assay in the presence of ETO with indicated concentration.(mean ± SD, n=3, *:p<0.05; ** :p<0.01; ***: p<0.001). **D**, etomoxir treatment interferes with Mutp53-inudced anchorage-independent growth. MCF12A-p53^{R273H} and MCF12A-p53^{R280K} were subjected to soft-agar foci formation assay in the presence of ETO with indicated amount for 21 days (mean ± SD, n=3). Statistic method: unpaired t-test, *:p<0.05; ** :p<0.01; ***: p<0.001.

Suppl. Fig. S3

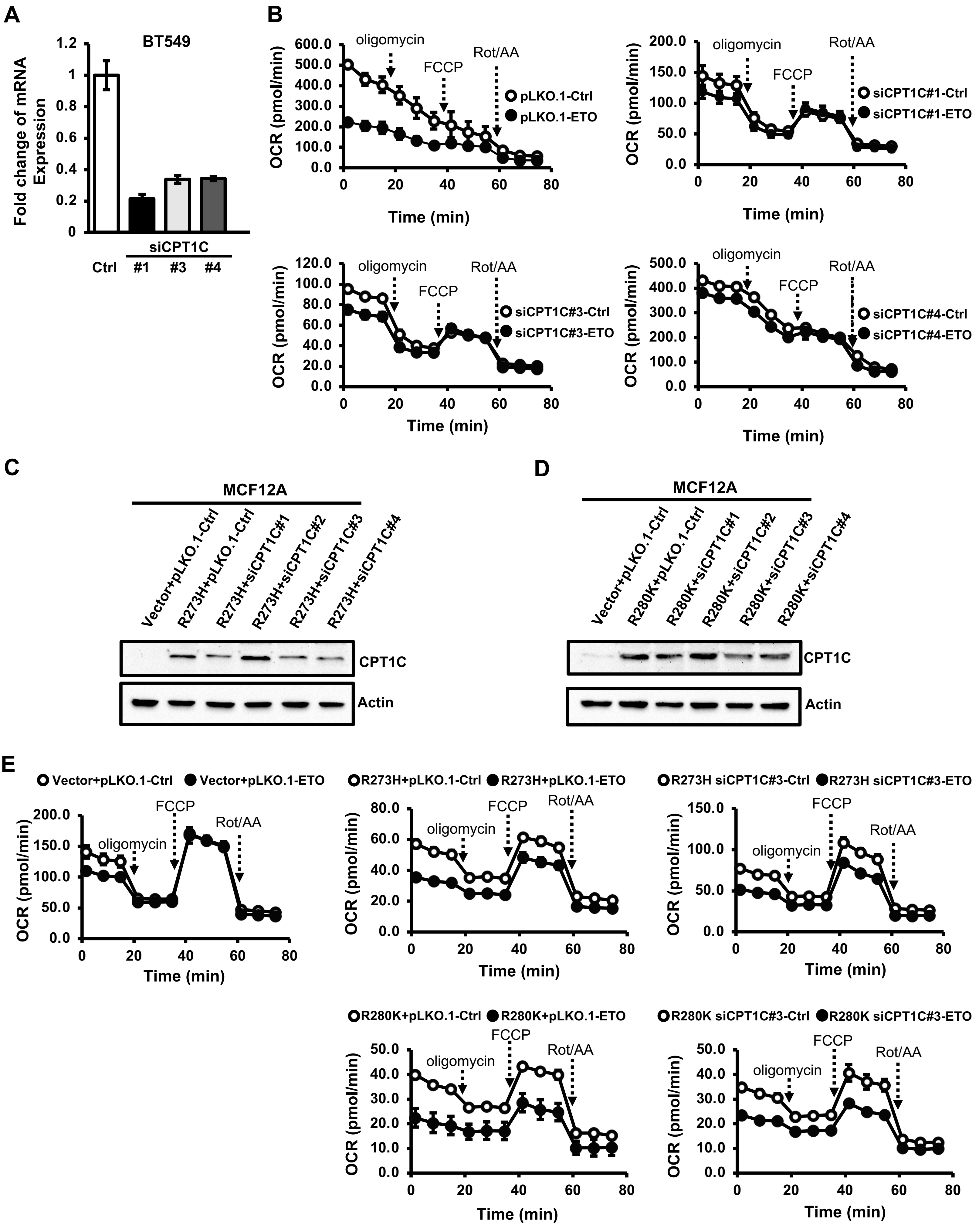
A
BT549
Fold change of mRNA Expression
1.2
1
0.8
0.6
0.4
0.2
0
siCPT1C
Ctrl
#1
#3
#4
B
oligomycin
FCCP
Rot/AA
OCR (pmol/min)
Time (min)
pLKO.1-Ctrl
pLKO.1-ETO
siCPT1C#1-Ctrl
siCPT1C#1-ETO
siCPT1C#3-Ctrl
siCPT1C#3-ETO
siCPT1C#4-Ctrl
siCPT1C#4-ETO
C
MCF12A
Vector+pLKO.1-Ctrl
R273H+pLKO.1-Ctrl
R273H+siCPT1C#1
R273H+siCPT1C#2
R273H+siCPT1C#3
R273H+siCPT1C#4
CPT1C
Actin
D
MCF12A
Vector+pLKO.1-Ctrl
R280K+pLKO.1-Ctrl
R280K+siCPT1C#1
R280K+siCPT1C#2
R280K+siCPT1C#3
R280K+siCPT1C#4
CPT1C
Actin
E
Vector+pLKO.1-Ctrl
Vector+pLKO.1-ETO
R273H+pLKO.1-Ctrl
R273H+pLKO.1-ETO
R273H siCPT1C#3-Ctrl
R273H siCPT1C#3-ETO
R280K+pLKO.1-Ctrl
R280K+pLKO.1-ETO
R280K siCPT1C#3-Ctrl
R280K siCPT1C#3-ETO
oligomycin
FCCP
Rot/AA
OCR (pmol/min)
Time (min)

Suppl. Fig. S3 (cont.)

Supplementary Figure S3. Knockdown of CPT1C decreases FAO activity in BLBC and mutant p53-bearing mammary epithelial cells.

A, the mRNA expression of *CPT1C* in CPT1C-KD BT549 cells. Relative mRNA expression of *CPT1C* was determined by Q-PCR (mean ± SD, n=3). **B**, the FAO activity is decreased by knockdown of CPT1C expression in BLBC cells. FAO activity of BT549-Ctrl, -siCPT1C#1, -siCPT1C#3,and -siCPT1C#4 were measured by seahorse metabolic flux analyzer with FAO assay kit. **C** and **D**, protein expression of CPT1C in CPT1C-KD MCF12A^{R273H} (**C**) and MCF12A^{R280K} (**D**). The expressional levels of CPT1C and Actin in control (Ctrl) and CPT1C-KD mutant p53-bearing MCF12A cells were examined by Western Blotting. **E**, downregulating CPT1C expression interferes with p53 mutation-enhanced FAO activity. FAO activity of MCF12A vector+pLKO.1-Ctrl, R273H/R280K+pLKO.1-Ctrl, and R273H/R280K-siCPT1C#3 were measured by seahorse metabolic flux analyzer with FAO assay kit.

Suppl. Fig. S4

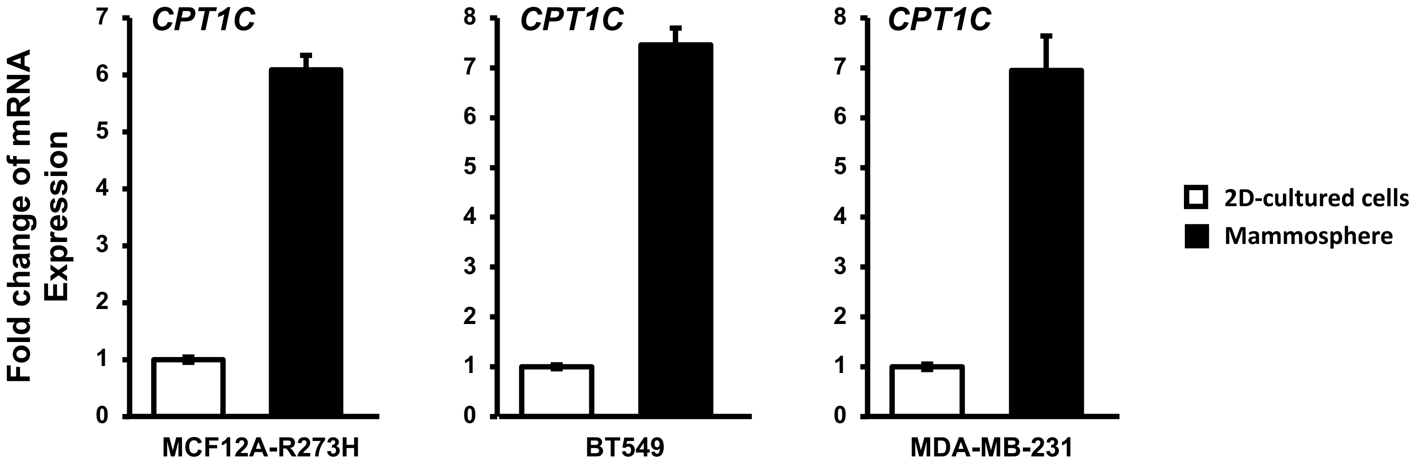

Supplementary Figure S4. The mRNA level of *CPT1C* is elevated in mammospheres.
The mRNA expression of *CPT1C* is upregulated in the primary mammopsheres from p53-R273H MCF12A, BT549, and MDA-MB-231 cells compared to the respective 2D-cultured cells. Relative mRNA expression of *CPT1C* between 2D-cultured cells and mammospheres were determined by Q-PCR (mean ± SD, n=3).

Suppl. Fig. S5

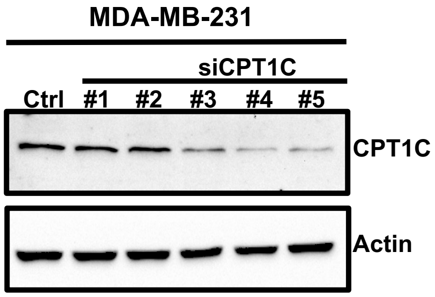

Supplementary Figure S5. Establishment of CPT1C-KD MDA-MB-231 cell lines.
The expressional levels of CPT1C and Actin in control (Ctrl) and CPT1C-KD MDA-MB-231 cells were examined by Western Blotting.

Suppl. Fig. S6

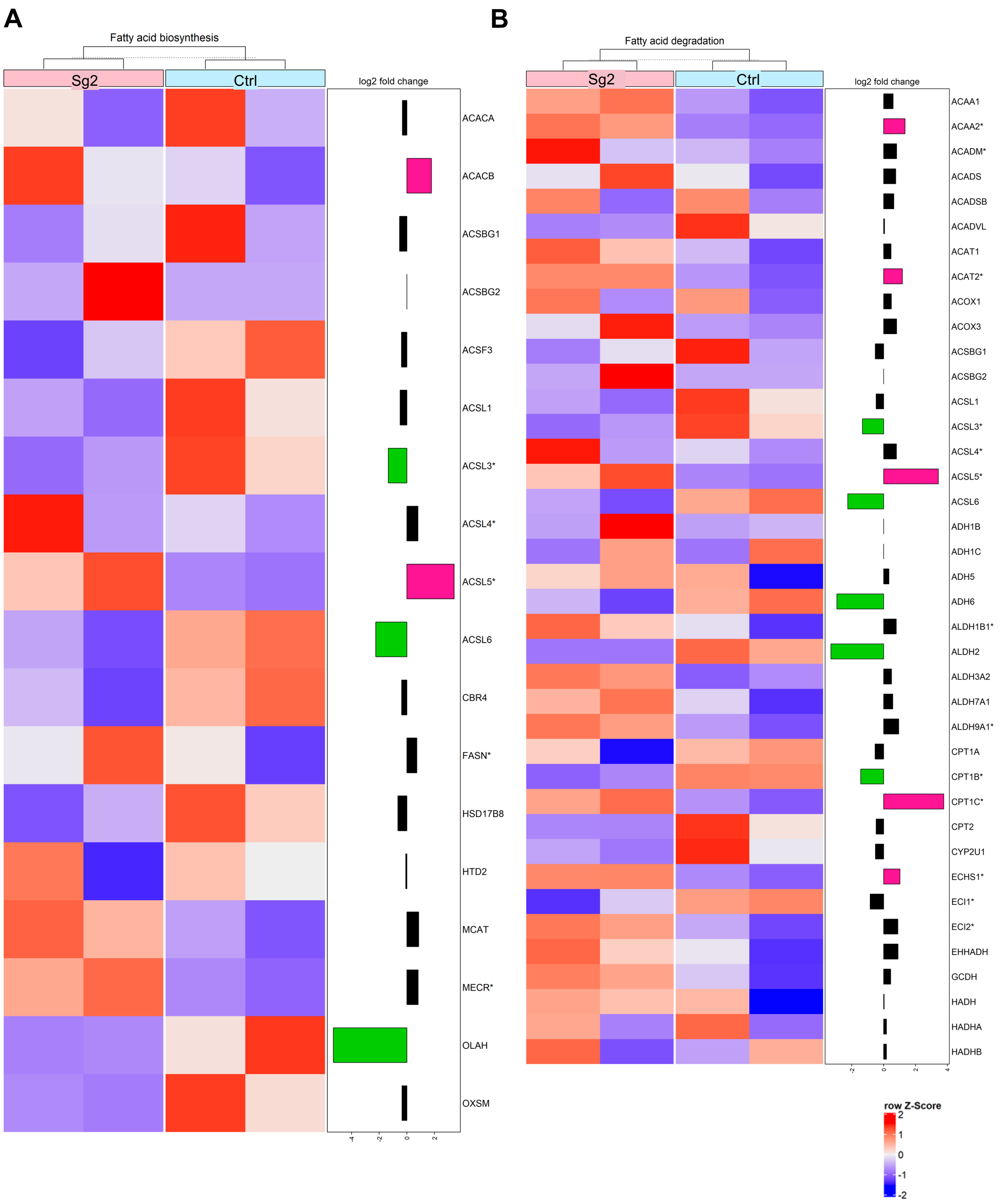

Supplementary Figure S6. miR-200c deficiency leads to altered expression of metabolic genes in lipid metabolism pathway.

A-B, heatmaps of regulatory genes in metabolic pathways including fatty acid biosynthesis (**A;** KEGG hsa00061), and fatty acid degradation (**B;** KEGG hsa00071).

Suppl. Fig. S7

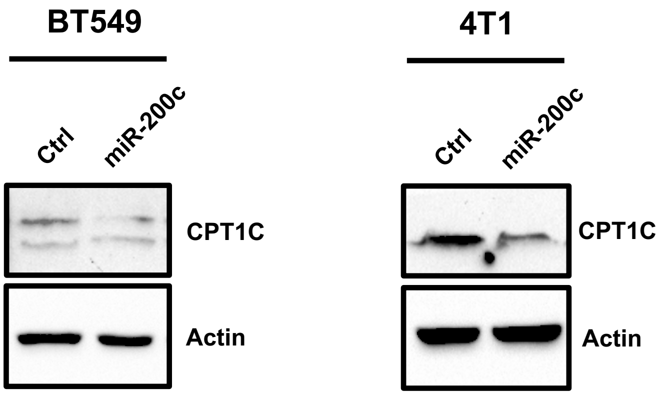

Supplementary Figure S7. Overexpression of miR-200c downregulates CPT1C in human BLBC cells bearing p53 mutations, and p53-null murine BLBC cells.

Restoration of miR-200c in BT549 and 4T1 cells decreases CPT1C expression. The expressional levels of CPT1C and Actin in control (Ctrl) and miR-200c-overexpressing cells were examined by Western Blotting.

Suppl. Fig. S8

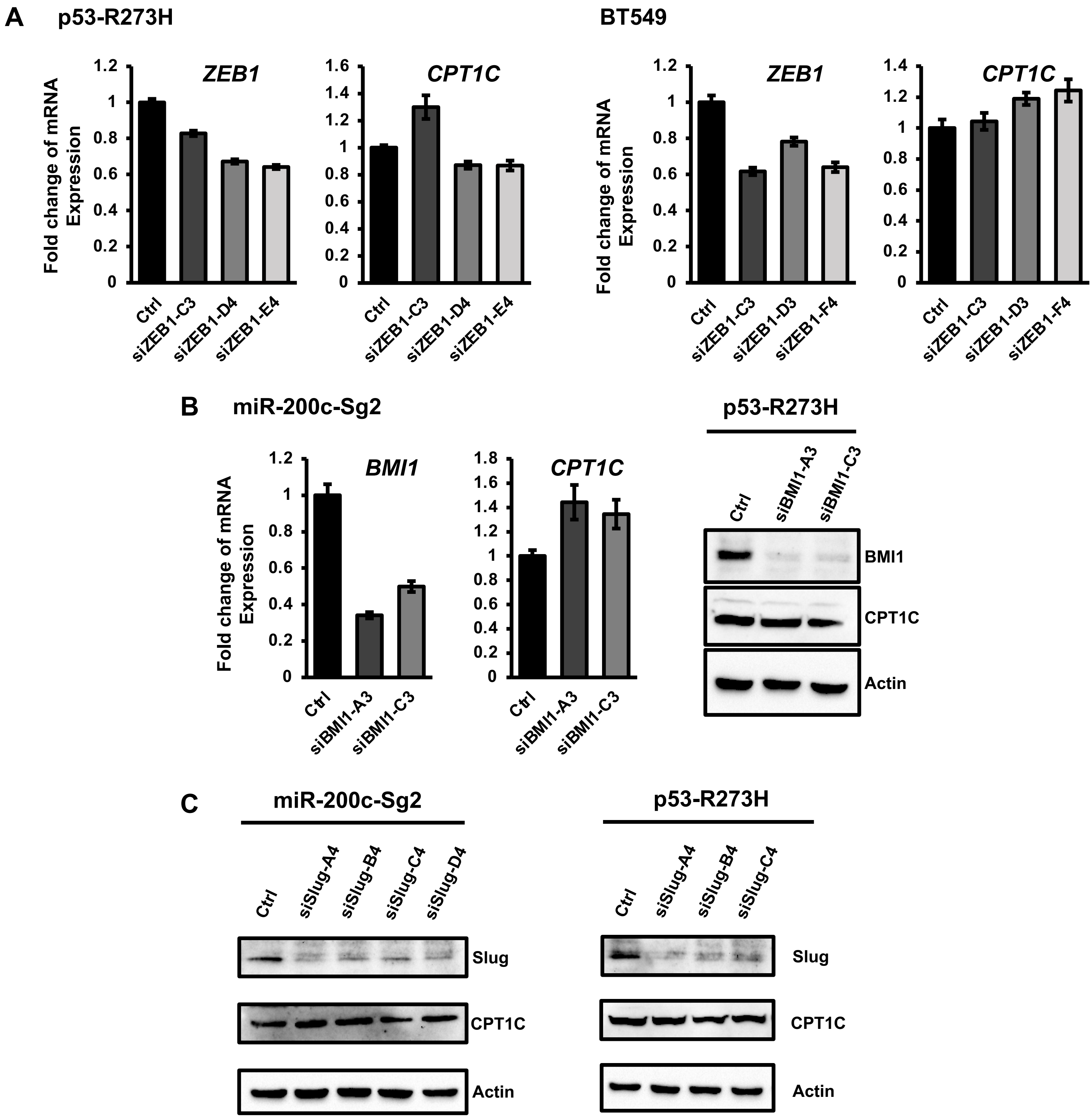

Supplementary Figure S8. CPT1C expression is not regulated by ZEB1, Bmi-1, or Slug in miR-200c-KO or p53 mutated mammary epithelial cells and basal-like breast cancer cells. Knockdown of ZEB1 (**A**), Bmi1 (**B**), and Slug (**C**) fail to affect CPT1C expression in miR-200C-KO (MCF12A miR-200c-Sg2), p53 mutant-overexpressing (MCF12A $p53^{R273H}$), or BT549 cells.

Suppl. Fig. S9

A

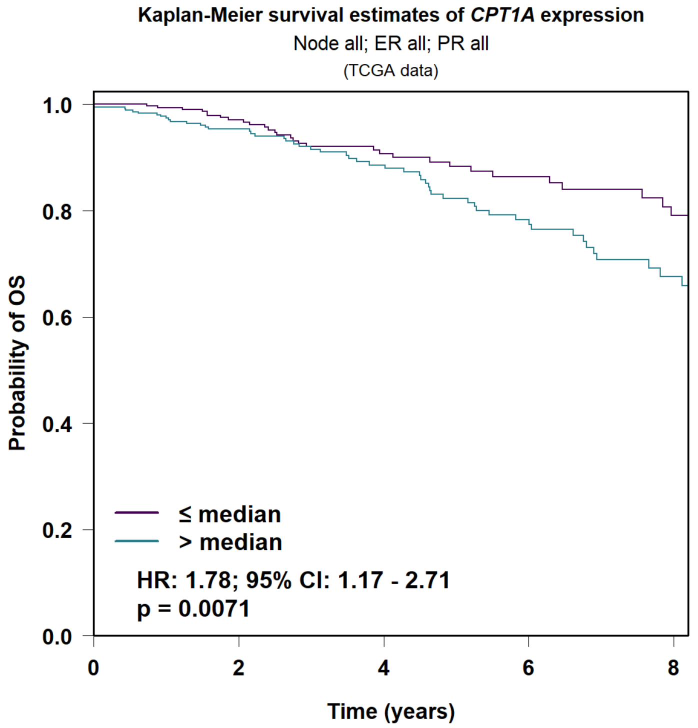

B

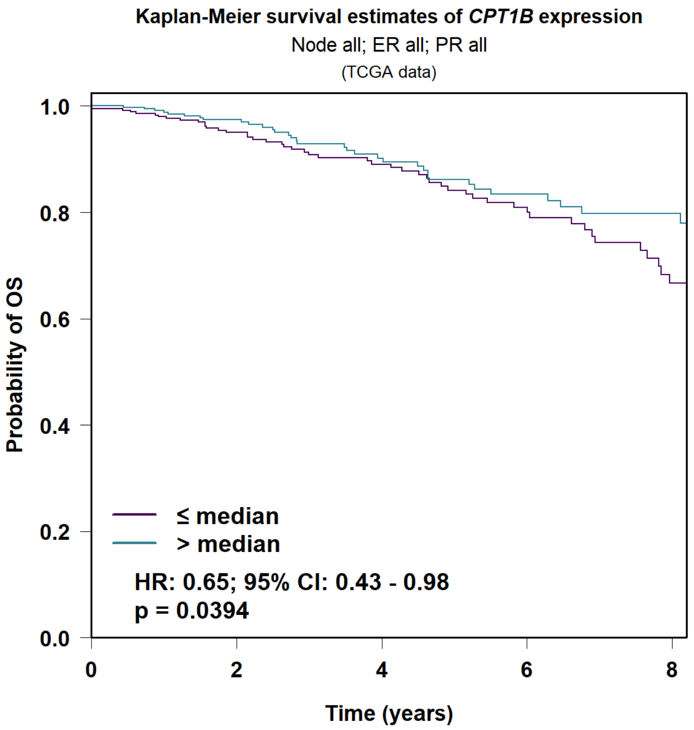

C

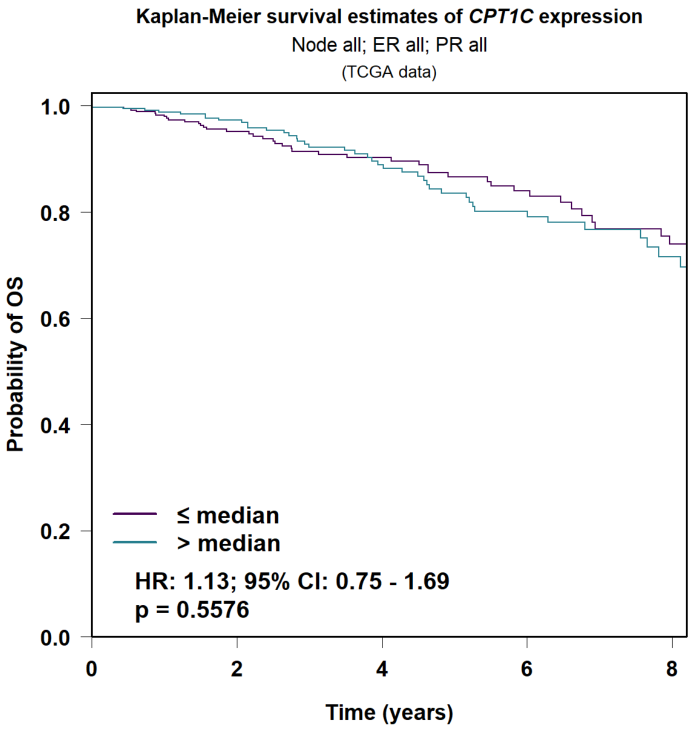

Supplementary Figure S9. High CPT1A expression, but not CPT1B or CPT1C, predicts a poor overall survival in total breast cancer patients in TCGA database. The Kaplan-Meier survival estimates of differential CPT1 expression were derived from the BLBC cohort of TCGA RNA-Seq data using bc-GeneExMiner v4.5 (http://bcgenex.ico.unicancer.fr) . The survival curve compared the patients with High (> median, green, n=371) and Low (≤median, purple, n=372) expression of CPT1A (**A**), CPT1B (**B**), or CPT1C (**C**). The statistical significance were determined with logrank test., p<0.05 is considered significant.

Suppl. Fig. S10

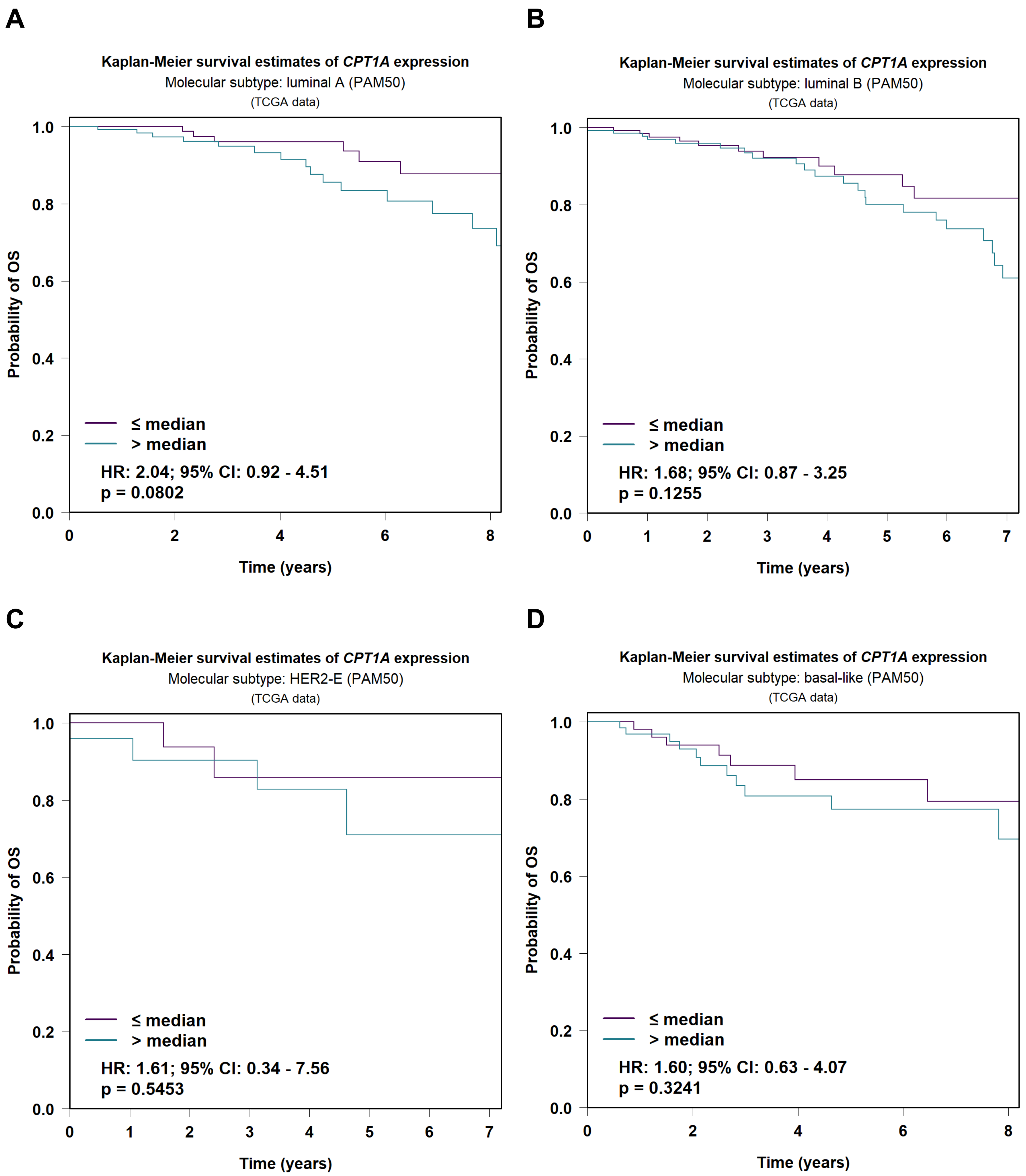

Supplementary Figure S10. High *CPT1A* expression shows a slight correlation with poor overall survival in luminal A breast cancer patients. The Kaplan-Meier survival estimates of differential *CPT1A* expression were derived from the BLBC cohort of TCGA RNA-Seq data using bc-GeneExMiner v4.5 (http://bcgenex.ico.unicancer.fr) . The survival curve compared the patients of luminal A (**A**), luminal B (**B**), Her2^{+} (**C**) or basal-like (**D**) subtype with High (> median, green) and Low (≤median, purple) expression of CPT1A. The statistical significance were determined with logrank test., p<0.05 is considered significant.

Suppl. Fig. S11

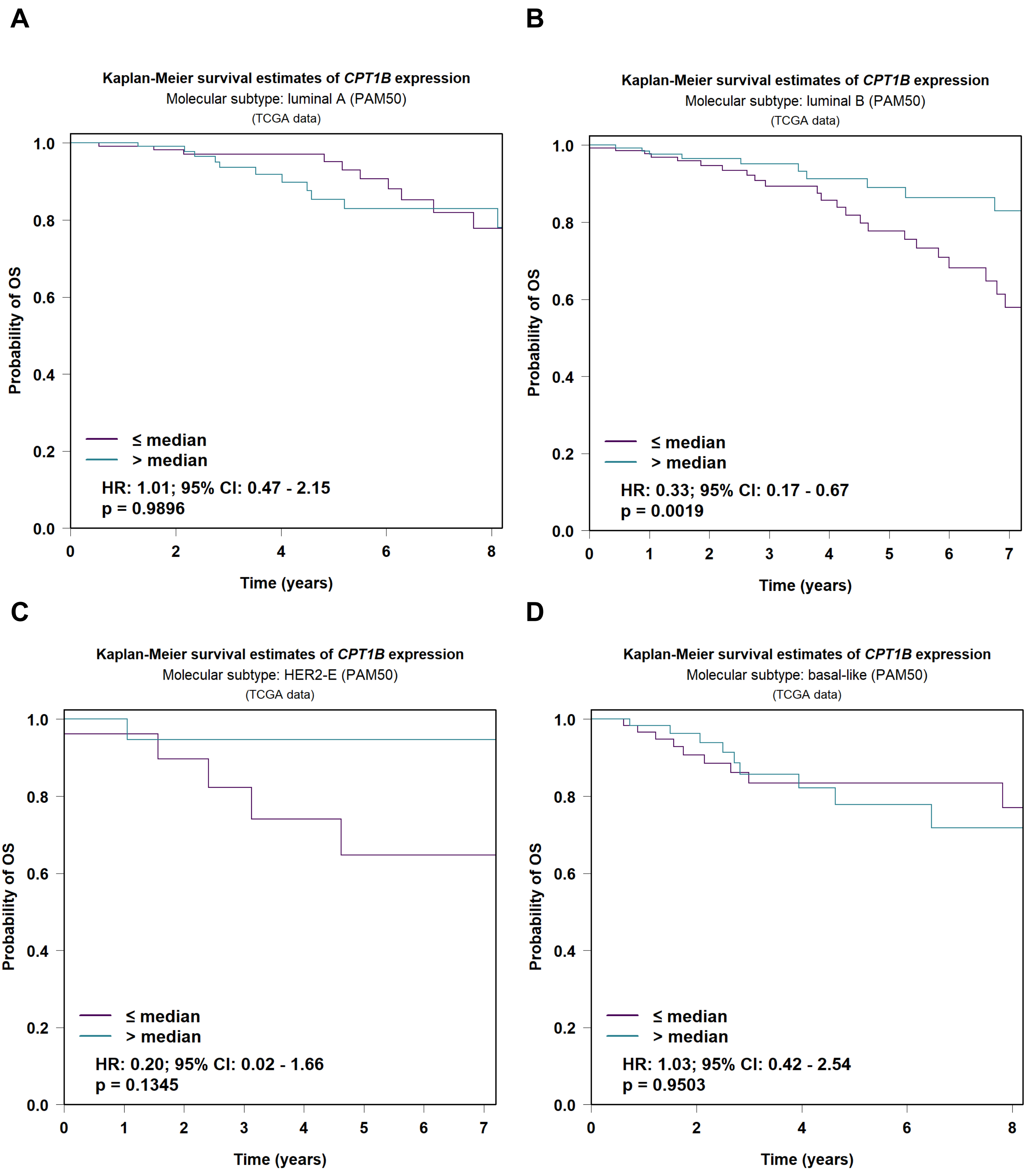

Supplementary Figure S11. High *CPT1B* expression predicts a better overall survival in luminal B breast cancer patients. The Kaplan-Meier survival estimates of differential *CPT1B* expression were derived from the BLBC cohort of TCGA RNA-Seq data using bc-GeneExMiner v4.5 (http://bcgenex.ico.unicancer.fr) . The survival curve compared the patients of luminal A (**A**), luminal B (**B**), Her2^{+} (**C**) or basal-like (**D**) subtype with High (> median, green) and Low (≤median, purple) expression of CPT1B. The statistical significance were determined with logrank test., p<0.05 is considered significant.

Suppl. Fig. S12

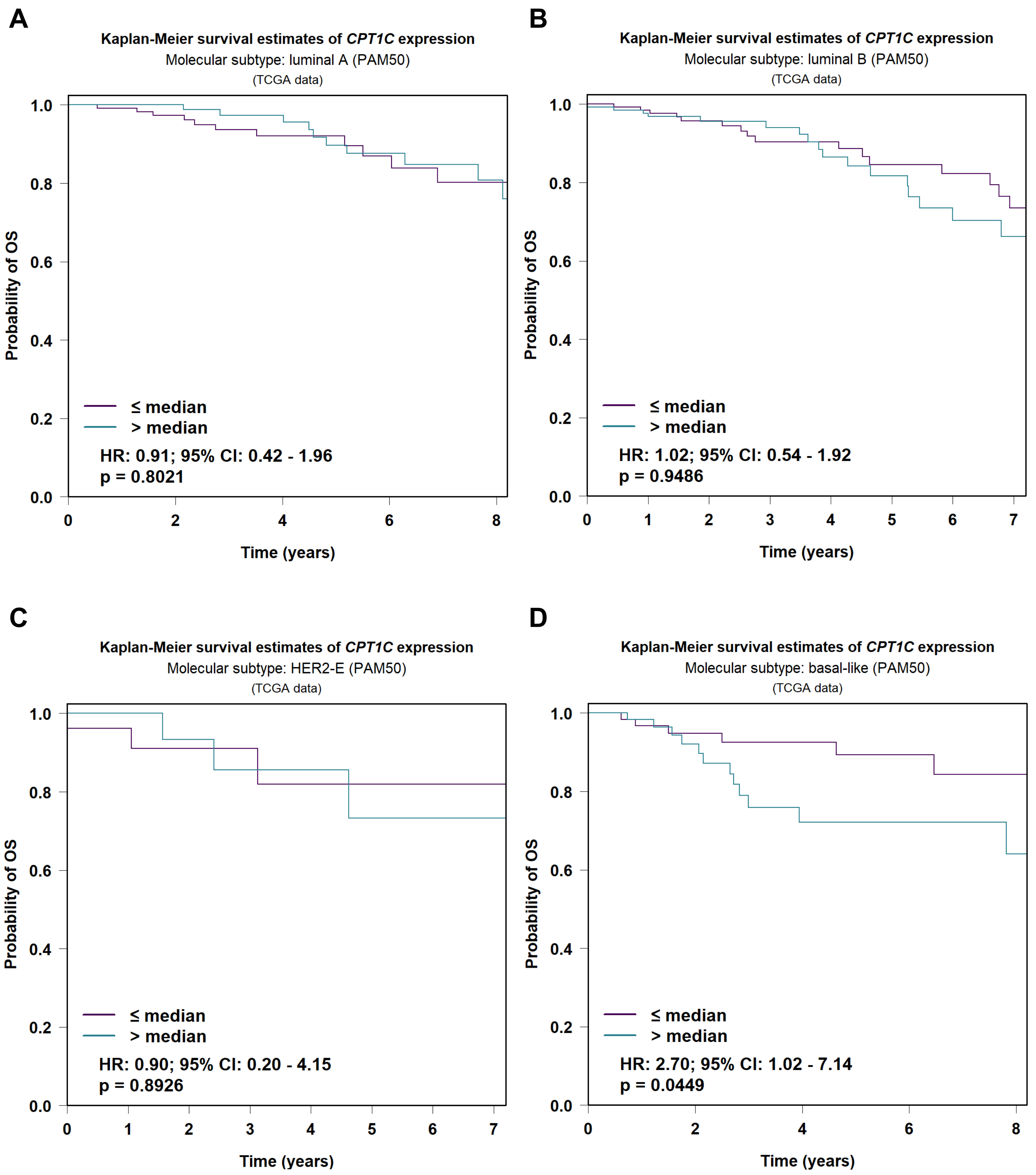

Supplementary Figure S12. High *CPT1C* expression predicts a poor overall survival in basal-like breast cancer patients. The Kaplan-Meier survival estimates of differential *CPT1C* expression were derived from the BLBC cohort of TCGA RNA-Seq data using bc-GeneExMiner v4.5 (http://bcgenex.ico.unicancer.fr) . The survival curve compared the patients of luminal A (**A**), luminal B (**B**), Her2^{+} (**C**) or basal-like (**D**) subtype with High (> median, green) and Low (≤median, purple) expression of CPT1C. The statistical significance were determined with logrank test., $p<0.05$ is considered significant.
