# Supplementary material for: Mutant p53-microRNA-200c-ZEB2-Axis-Induced CPT1C Elevation Contributes to Metabolic Reprogramming and Tumor Progression in Basal-Like Breast Cancers
Source: Front Oncol. 2022 Jul 21;12:940402. doi: 10.3389/fonc.2022.940402 (PMC9351713; doi:10.3389/fonc.2022.940402)
Supplement: Supplementary file 2 [file DataSheet_2.pdf]

Mutant p53-microRNA-200c-ZEB2-axis-induced CPT1C Elevation Contributes to Metabolic Reprogramming and Tumor Progression in Basal-like Breast Cancers

Chen-Yun Wang[2,3#], Cing-Hong Wang[2,3#], Ru-Tsun Mai[1,2,3#], Ting-Wen Chen[1,3,4], Chia-Wei Li[5], and Chi-Hong Chao[1,2,3]*

Supplementary Materials and Methods

FACS analysis

Cells were stained with FITC-conjugated anti-CD44 antibody (BD Biosciences) and PE-conjugated anti-CD24 antibody (BD Biosciences) then subjected to FACS analysis by NovoCyte Flow Cytometer (ACEA Biosciences).

RNA sequencing data availability and analysis

The public RNA-Seq data of miR-200c KO MCF12A cells are available under BioProject ID: PRJNA683086. (https://www.ncbi.nlm.nih.gov/Traces/study/?acc1⁄4PRJNA683086). Detailed analytic procedures were described in our previous study (1).

References

1. Chao C-H, Wang C-Y, Wang C-H, Chen T-W, Hsu H-Y, Huang H-W, et al. Mutant p53 attenuates oxidative phosphorylation and facilitates cancer stemness through downregulating miR-200c–PCK2 axis in basal-like breast cancer. Mol Cancer Res. 2021;19(11):1900-16.
